# A polymeric nanocarrier that eradicates breast cancer stem cells and delivers chemotherapeutic drugs

**DOI:** 10.1186/s40824-023-00465-9

**Published:** 2023-12-15

**Authors:** Li Lv, Yonghui Shi, Zhicheng Deng, Jiajia Xu, Zicong Ye, Jianxiong He, Guanghui Chen, Xiaoxia Yu, Junyan Wu, Xingzhen Huang, Guocheng Li

**Affiliations:** 1 Department of Pharmacy, Guangdong Provincial Key Laboratory of Malignant Tumor Epigenetics and Gene Regulation, Sun Yat-Sen Memorial Hospital, Sun Yat-Sen University, Guangzhou, Guangdong 510120 China; 2 Shenshan Medical Center, Memorial Hospital of Sun Yat-Sen University, Shanwei, Guangdong 516600 China; 3 School of Pharmacy, Guangxi Medical University, Nanning, Guangxi 530021 China

**Keywords:** Nanocarrier, Breast cancer stem cell, Chemotherapy, Breast cancer, Amphiphilic polymer

## Abstract

**Background:**

Drug nanocarriers can markedly reduce the toxicities and side effects of encapsulated chemotherapeutic drugs in the clinic. However, these drug nanocarriers have little effect on eradicating breast cancer stem cells (BCSCs). Although compounds that can inhibit BCSCs have been reported, these compounds are difficult to use as carriers for the widespread delivery of conventional chemotherapeutic drugs.

**Methods:**

Herein, we synthesize a polymeric nanocarrier, hyaluronic acid-block-poly (curcumin-dithiodipropionic acid) (HA-b-PCDA), and explore the use of HA-b-PCDA to simultaneously deliver chemotherapeutic drugs and eradicate BCSCs.

**Results:**

Based on molecular docking and molecular dynamics studies, HA-b-PCDA delivers 35 clinical chemotherapeutic drugs. To further verify the drug deliver ability of HA-b-PCDA, doxorubicin, paclitaxel, docetaxel, gemcitabine and camptothecin are employed as model drugs to prepare nanoparticles. These drug-loaded HA-b-PCDA nanoparticles significantly inhibit the proliferation and stemness of BCSC-enriched 4T1 mammospheres. Moreover, doxorubicin-loaded HA-b-PCDA nanoparticles efficiently inhibit tumor growth and eradicate approximately 95% of BCSCs fraction in vivo. Finally, HA-b-PCDA eradicates BCSCs by activating Hippo and inhibiting the JAK2/STAT3 pathway.

**Conclusion:**

HA-b-PCDA is a polymeric nanocarrier that eradicates BCSCs and potentially delivers numerous clinical chemotherapeutic drugs.

**Graphical Abstract:**

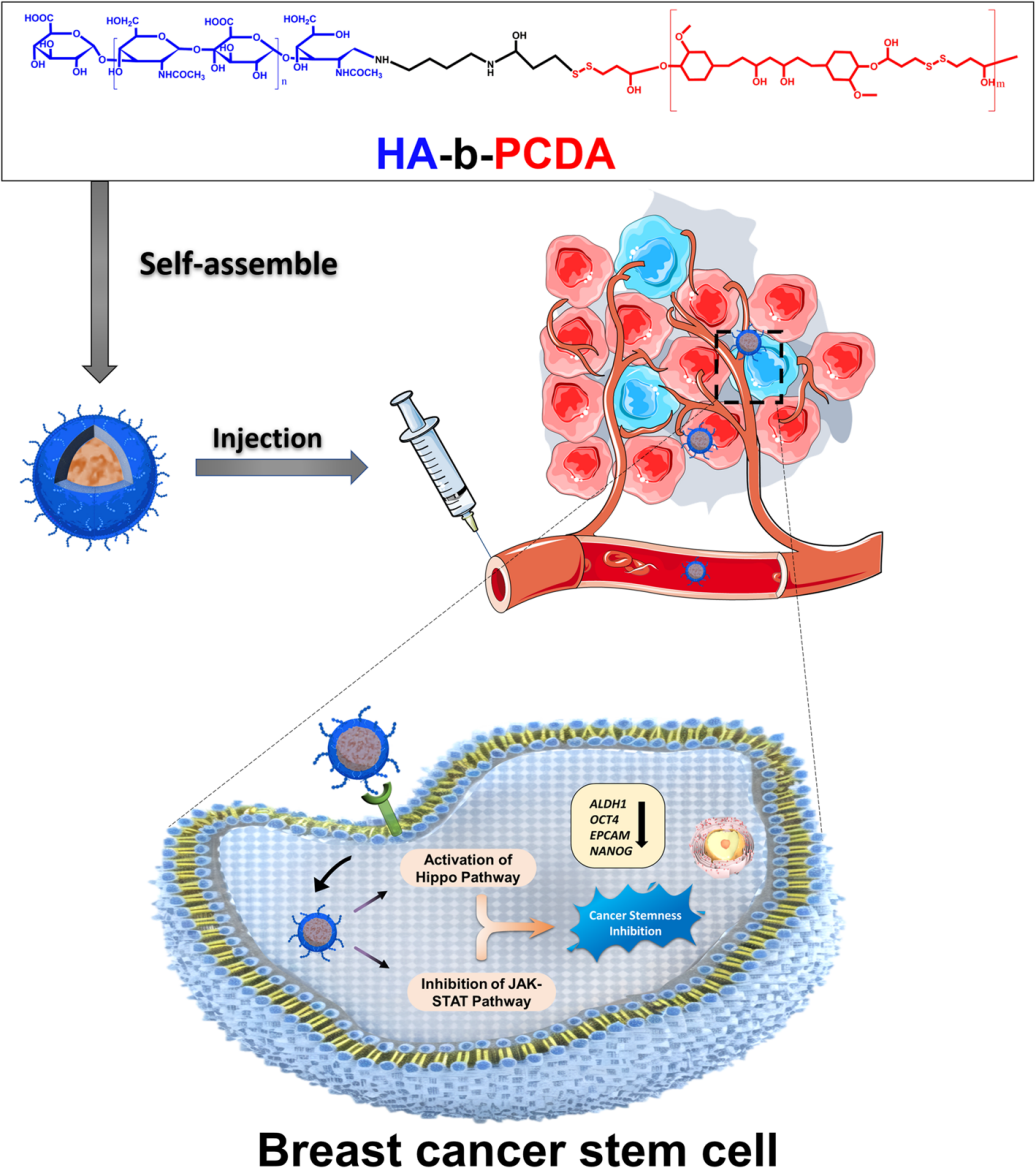

**Supplementary Information:**

The online version contains supplementary material available at 10.1186/s40824-023-00465-9.

## Background

Currently, chemotherapy is the standard treatment for patients with triple negative breast cancer [1–3]. Unfortunately, conventional chemotherapeutic drugs have several limitations, such as serious toxicities and side effects caused by nonspecific biodistribution, poor tumor accumulation, and rapid blood/renal clearances in clinical settings. Remarkable efforts have been made to overcome these limitations. Nanocarriers, including polymeric nanoparticles (NPs) [4], liposomes [5], dendrimers NPs [6], albumin NPs [7], and inorganic NPs [8], have excellent advantages [9–11]. Nanocarriers enhance the accumulations of encapsulated chemotherapeutic drugs in tumor tissues through the enhanced permeability and retention (EPR) effect and active targeting effect mediated by receptors overexpressed on the tumor cell surface. In addition, nanocarriers prolong the blood circulation time of drugs and reduce systemic toxicity and side effects by minimizing the drug distribution in normal tissues and organs [12–19]. Among the nanocarriers, polymeric NPs and liposomes are being intensively studied due to their good biocompatibility [5, 20–23]. Polymeric NPs and liposomes that are commercially available include Doxil® [24], Myocet® [11], Lipusu® [25], Genexol-PM® [26], and Nanoxel® [27].

Although polymeric NPs and liposomes could significantly reduce the toxicities and side effects of encapsulated drugs in the clinic, tumor recurrence and drug resistance still occur in patients adminstrated these drug nanocarriers. Based on compelling evidence, residual tumors after clinical treatment with drug nanocarriers or free drugs can enrich breast cancer stem cells (BCSCs), leading to the recurrence and drug resistance of tumors [28–30]. BCSCs are a rare population of cancer cells that overexpress the cluster of differentiation 44 (CD44) receptor [31] and aldehyde dehydrogenase 1 (ALDH1) [32]. Conventional chemotherapeutic drugs can not eliminate cancer stem cells (CSCs), despite their ability to kill cancer cells and shrink tumor size. Residual CSCs can lead to tumor recurrence through self-renewal and differentiation into multiple cancer cell types. Drug nanocarriers which have been used in clinic have little effect on eradicating BCSCs because they are prepared by encapsulating chemotherapeutic drugs with carriers lacking biological activity. Notably, biodegradable polymeric carriers that can eradicate CSCs have rarely been reported. However, certain compounds have been reported to inhibit CSCs, including curcumin (CUR) [33, 34], salinomycin [35], 8-hydroxyquinoline [36], and thioridazine hydrochloride [37]. Among these compounds, the natural phenolic compound CUR has the lowest toxicity and fewest side effects. Based on prior reports, daily administration of 12 g of CUR is very safe for intake [38]. Therefore, CUR has highly biological safety and is widely used as a food additive. Although certain agents can inhibit CSCs, these cells can spontaneously and stochastically originate from non-CSCs [39]. Therefore, treatments targeting only CSCs might not cure cancer, and simultaneous eradication of CSCs and non-CSCs is critical for effective cancer therapy. However, most reported compounds that detrimentally affect CSCs are small molecular compounds that are difficult to use as carriers to encapsulate chemotherapeutic drugs and achieve coordinated anticancer effects. Thus, the construction of a polymeric nanocarrier that can eradicate BCSCs and deliver chemotherapeutic drugs to simultaneously eliminate breast cancer cells (BCCs) and BCSCs is needed.

**Scheme 1:**
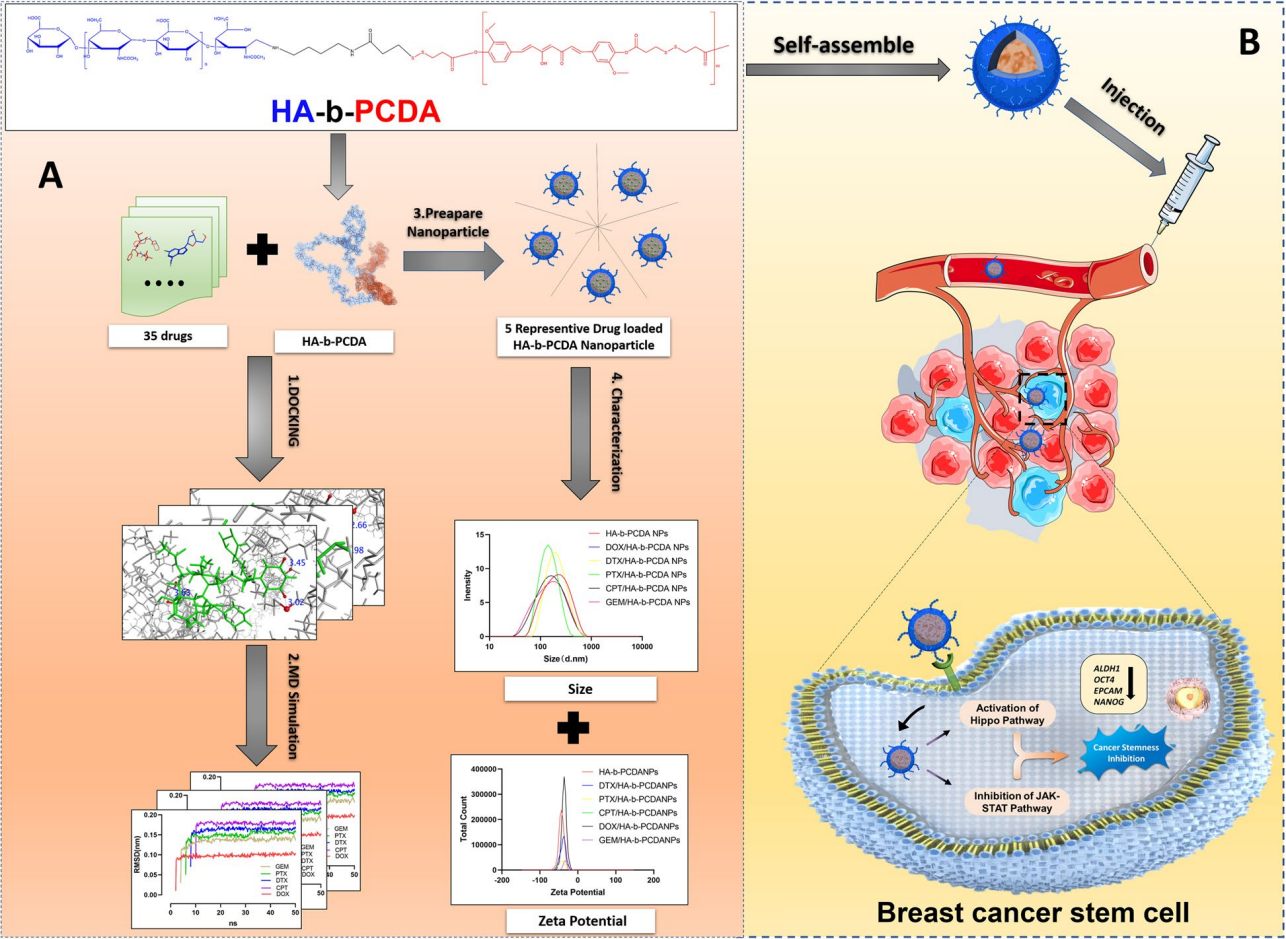
HA-b-PCDA as a nanocarrier to deliver drugs and eradicate BCSCs. **A** HA-b-PCDA can intact with 35 common clinical chemotherapeutic drugs to form stable complexes verifying by the molecular docking and molecular dynamics study, and represented drug-loaded HA-b-PCDA nanoparticles were prepared and characterized. **B** HA-b-PCDA could activate the Hippo pathway and inhibit the JAK2/STAT3 pathway to eradicate BCSCs

In this study, we synthesized an amphiphilic polymer, hyaluronic acid-block-poly (curcumin-dithiodipropionic acid) (HA-b-PCDA), using HA as the hydrophilic block and PCDA, which was previously synthesized by our group, as the hydrophobic block (Scheme 1) [40]. PCDA is synthesized by polymerizing CUR, a relatively non-toxic compound for the human body. Unlike other biologically inert polymer carriers such as poly(ε-caprolactone), poly (lactic-co-glycolic acid) and polylactic acid, PCDA offers advantage as a drug delivery system because it can encapsulate chemotherapy drugs while having potential anti-CSC properties. CD44 is a transmembrane proteoglycan that is overexpressed in many cancer cell types, including CSCs. This makes it an appealing target for active delivery of drug carriers [41–44]. CD44-targeted drug delivery systems can selectively transport therapeutics to cancers and CSCs while minimizing effects on normal cells [41, 43]. HA is a natural CD44 ligand that can be used to modify drug carriers, conferring CD44-targeting ability along with biodegradability and biocompatibility for safe use in humans [41, 44]. Overall, HA-modified delivery systems leverage CD44 overexpression in cancers to achieve active targeting of chemotherapeutics specifically to non-CSC and CSC populations. We hypothesized that the synthesized HA-b-PCDA could deliver numerous clinical chemotherapeutic drugs and eradicate BCSCs. To prove that HA-b-PCDA could be widely used as a nanocarrier to deliver numerous clinical chemotherapeutic drugs, molecular docking and molecular dynamics were first performed to investigate the capability of HA-b-PCDA to deliver 35 clinical chemotherapeutic drugs. Thereafter, HA-b-PCDA NPs loaded with doxorubicin (DOX/HA-b-PCDA NPs), paclitaxel (PTX/HA-b-PCDA NPs), docetaxel (DTX/HA-b-PCDA NPs), gemcitabine (GEM/HA-b-PCDA NPs), and camptothecin (CPT/HA-b-PCDA NPs) were prepared and characterized to further verify the drug deliverability of HA-b-PCDA. DOX/HA-b-PCDA NPs were selected for further studies as these NPs are expected to accumulate in tumor tissues based on the EPR and active targeted effects mediated by CD44 receptors overexpressed on the surfaces of BCCs and BCSCs and eradicate both BCCs and BCSCs. Thus, to verify that DOX/HA-b-PCDA NPs could be a powerful platform for simultaneously targeting BCCs and BCSCs, we evaluated the cellular uptake and cytotoxicity of DOX/HA-b-PCDA NPs in 4T1 BCCs and BCSC-enriched 4T1 mammosphere cells. The effects of DOX/HA-b-PCDA NPs on disrupting existing BCSC-enriched 4T1 mammospheres and prevention of secondary BCSC-enriched 4T1 mammosphere formation were evaluated. In addition, it was also investigated the effect of DOX/HA-b-PCDA NPs on the eradicating the ALDH^high^ cell portion in BCSC-enriched 4T1 mammospheres. The in vivo biodistribution and antitumor efficacy of DOX/HA-b-PCDA NPs were evaluated in a 4T1-subcutaneous mice model. Finally, the underlying molecular mechanism of HA-b-PCDA in eradicating BCSCs was explored using transcriptome sequencing, quantitative real-time polymerase chain reaction (RT-qPCR) and western blot (WB).

## Methods

### Materials

Doxorubicin hydrochloride, triethylamine and dimethyl sulfoxide (DMSO) were purchased from Sigma–Aldrich Chemicals (St. Louis, Mo., USA). GEM, PTX, DTX, CPT and 1,1'-dioctadecyl-3,3,3',3'-tetramethylindotricarbocyanine iodide (Dir) were obtained from Meilunbio, Dalian, China. Dialysis bag were obtained from Solarbio Life Sciences, Beijing, China. Penicillin/streptomycin were purched from Gibco, Invitrogen, Herlev, Denmark.

### Molecular docking

The 3D structure of HA-b-PCDA was generated, and energy minimization was performed using molecular operating environment (MOE 2019, Chemical Computing Group, Canada). The drug structures were downloaded from the ChemSpider website (http://www.chemspider.com). Molecular docking studies were performed using AutoDock Vina (version 1.1.2) with the polymer as the receptor and the drugs as ligands [45, 46]. The search grid for HA-b-PCDA was identified using the X, Y, and Z centers of 200, 10, and -6. The sizes of X, Y, and Z are 39.76, 24.14, and 62.48, respectively. The binding energy of the drug to HA-b-PCDA was calculated using the AutoDock Vina scoring function.

### All-atom molecular dynamics study

The complex structures with the lowest affinity from the results of molecular docking were used for all-atom molecular dynamics study [47, 48]. Simulations were performed under the all-atom optimized potentials for liquid simulations (OPLS-AA) force field in the 2019 MOE [49, 50]. The initial structures of HA-b-PCDA and drug complex were solvated with 10000 module waters. During the generation of applicable geometry molecular dynamics simulation systems, the complex in solvation was processed for energy minimization using the steepest-descent procedure at 0.1 root-mean-square (RMS) kcal/mol/A^2^. Subsequently, temperature (300 K) and pressure (1 bar) stabilization were predicted using the equilibrium stage (100 ps), respectively. With a production stage of 50 ns, every trajectory view was proceeded at 0.002 ps for each time step and recorded every 20 ps for a frame. Molecular dynamic results were analyzed according to trajectory and depicted as a plot with root-mean-square deviation (RMSD) using the Visual Molecular Dynamics (VMD, 1.9.4) software [46, 51].

### Fabrication and characterization of NPs

DOX/HA-b-PCDA NPs were prepared using a modified solvent-dialysis method as previously reported [52]. Briefly, HA-b-PCDA (20 mg), doxorubicin hydrochloride (2 mg), and triethylamine (20 μL) were added to DMSO (4 mL) with stirring. Ultrapure water (40 mL) was slowly added to the mixed solution, which was stirred for 15 min. Subsequently, the mixed solution was dialyzed against ultrapure water for 12 h to remove the organic solvents. After dialysis, the mixed solution was centrifuged at 3,000 rpm for 15 min to remove the unloaded DOX and large particles, forming a solution of DOX/HA-b-PCDA NPs. HA-b-PCDA NPs were prepared using the same method without adding DOX hydrochloride and triethylamine. PTX/HA-b-PCDA NPs, DTX/HA-b-PCDA NPs, GEM/HA-b-PCDA NPs and CPT/HA-b-PCDA NPs were prepared using the same method by replacing DOX hydrochloride with PTX, DTX, and CPT without the addition of triethylamine. The size distribution and zeta potential of the prepared NPs were characterized using a ZS90 dynamic-light scattering analyzer (Malvern Panalytical, Malvern, UK). The morphology of DOX/HA-b-PCDA NPs was observed using transmission electron microscopy—JEM-2010HT instrument (JEOL, Tokyo, Japan).

### Encapsulation efficiency and drug loading content of NPs

NPs were destroyed in methanol and ultrasonicated for 10 min. The drug content in the NPs was determined using liquid chromatography tandem mass spectrometry (LC–MS/MS). The encapsulation efficiency and drug loading content of the NPs were calculated using the following equations:

$$\mathrm{Drug\ loading\ content}\left(\mathrm{\%}\right)=\frac{\mathrm{drug\ content\ in\ NPs}}{\mathrm{mass\ of\ NPs}}\times 100\mathrm{\%}$$

$$\mathrm{Encapsulation\ Efficiency}\left(\mathrm{\%}\right)=\frac{\mathrm{drug\ content\ in\ NPs}}{\mathrm{drug\ feeding}}\times 100\mathrm{\%}$$

### In vitro drug release

A suspension of DOX/HA-b-PCDA NPs (5 mL) was added to a dialysis bag with a molecular weight cutoff of 3500, which was then immersed in PBS (15 mL, pH 7.4) containing 1% Tween 80 (v/v) with stirring at 150 rpm at 37 °C. At predetermined time points, 1 mL of external release medium was collected and replaced with fresh medium. The amount of DOX released from the NPs was determined using fluorescence spectrophotometry at excitation and emission wavelengths of 496 and 592 nm, respectively.

### Culture of 4T1 BCCs

4T1 BCCs obtaubed from Shanghai Cell Bank, Chinese Academy of Sciences were cultured in roswell park memorial institute (RPMI) 1640 medium supplied with 10% fetal bovine serum (Gibco, Franklin Lakes, NJ, USA), 100 U/mL of penicillin and 100 μg/mL of streptomycin at 37 °C with 5% CO_2_ in a humidified incubator.

### Suspension culture for the formation of BCSC-enriched 4T1 mammospheres

4T1 BCCs were cultured in 6-well ultralow attachment plates at a density of 2 × 10^5^ cells per well with serum-free dulbecco's modified eagle's medium (DMEM)/F12 basal culture medium supplemented with 40 μg/mL epidermal growth factor (Beyotime, Jiangsu, China), 40 μg/mL basic fibroblast growth factor (bFGF), 0.4% (v/v) bovine serum albumin (Beyotime), 5 μg/mL insulin (Yuanyebio, Shanghai, China), 100 U/mL of penicillin and 100 μg/mL of streptomycin. After 7 d of culture, BCSC-enriched 4T1 mammospheres were collected for further experiments.

### Characterization of the BCSC-enriched 4T1 mammospheres

To characterize the stemness of BCSC-enriched 4T1 mammospheres, the expression levels of typical BCSC markers ALDH1, CD44, and CD24 were quantified using flow cytometry FACSCalibur device (BD, San Jose, CA, USA). The mammospheres were collected via centrifugation and dissociated using trypsin/EDTA. Dissociated single mammosphere cells were stained with ALDEFLUOR Kit (STEMCELL Technologies, Vancouver, BC, Canada) to quantify the proportion of ALDH^high^ cells in the mammospheres. To detect the proportion of CD44^+^/CD24^−/low^ cells in the mammoshperes, the cells were stained with the primary antibodies CD44-FITC (Invitrogen, Carlsbad, CA, USA) and CD24-PE (Invitrogen), according to the manufacturer’s instructions. For comparison, the expression levels of ALDH1, CD44, and CD24 in 4T1 BCCs were determined.

### In vitro cellular uptake

To evaluate the accessibility of DOX/HA-b-PCDA NPs to BCCs and BCSCs, the cellular uptake of DOX/HA-b-PCDA NPs and free DOX (control group) by 4T1 BCCs, noncancerous 293 T cells, and BCSC-enriched 4T1 mammosphere cells was determined using microscopy and flow cytometry. For microscopy, cells were seeded in 15 mm confocal imaging dishes (5 × 10^5^ cells per dish) and cultured overnight. DOX/HA-b-PCDA NPs or free DOX was added at a DOX concentration of 5 μg/mL and incubated for 4 h. Subsequently, the drug-containing medium was removed, and the cells were rinsed three times with PBS and fixed with 4% paraformaldehyde. The fixed samples were stained with DAPI (5 μM) and observed under fluorescence microscopy with an Imager A2 instrument (Carl Zeiss, Jena, Germany) or via confocal laser scanning microscope (CLSM) using an LSM 800 instrument with Airyscan (Carl Zeiss). For flow cytometry analysis, the cells were seeded in 24-well plates at a density of 5 × 10^4^ cells per well and cultured overnight. DOX/HA-b-PCDA NPs or free DOX was added at a DOX concentration of 5 μg/mLand incubated for 4 h. The treated cells from each well were collected using trypsin and centrifuged at 1,000 rpm for 5 min. The collected cells were resuspended in PBS (0.5 mL) and analyzed using flow cytometry (FACSCalibur, BD). For the competitive inhibition experiment, the cells were pre-incubated with 1 mM HA for 1 h. DOX/HA-b-PCDA NPs or free DOX were added, and the cells were incubated for 4 h. The following steps were repeated as described above.

To observe the penetration of DOX/HA-b-PCDA NPs in BCSC-enriched 4T1 mammospheres, the collected mammospheres were seeded in 6-well ultralow attachment plates and incubated with DOX/HA-b-PCDA NPs or free DOX at a DOX concentration of 5 μg/mL for 4 h. The fixation, staining and observation procedures were the same as described above. To evaluate the accessibility of DOX/HA-b-PCDA NPs to ALDH1^high^ or ALDH1^low^ cells of BCSC-enriched 4T1 mammospheres, DOX/HA-b-PCDA NPs or free DOX were incubated with BCSC-enriched 4T1 mammospheres at a DOX concentration of 5 μg/mL for 4 h. The mammospheres were then dissociated into single cells using trypsin. Single cells were collected and stained with Aldefluor fluorescent reagent, and DOX signals in ALDH1^high^, ALDH1^low^ cells and total cells of BCSC-enriched 4T1 mammospheres were measured using flow cytometry.

### MTT assay

To assess the in vitro selectivity of the anti-BCC ability of NPs, the cytotoxicity of DOX/HA-b-PCDA NPs or free DOX was evaluated using 4T1 BCCs and noncancerous 293 T cells. Briefly, cells were seeded in normal 96-well culture plates at a density of 5000 cells per well and incubated overnight. DOX/HA-b-PCDA NPs or free DOX were added to each well at different concentrations and incubated for another 72 h. After incubation, cell viability was determined using the standard MTT assay.

### CCK-8 assay

To evaluate the in vitro anti-BCSC ability of the NPs, the cytotoxicity of DOX/HA-b-PCDA NPs or free DOX against BCSC-enriched 4T1 mammosphere cells was evaluated. Cells were seeded into ultralow attachment 96-well plates at a density of 5,000 cells per well and incubated overnight. Subsequently, BCSC-enriched 4T1 mammosphere cells were treated with DOX/HA-b-PCDA NPs or free DOX at different concentrations for 72 h. The viability of the BCSC-enriched 4T1 mammosphere cells was estimated using the CCK-8 assay.

### Cell apoptosis

To determine the apoptotic rate of BCCs after different treatments, 4T1 BCCs were seeded in a 6-well plate at a density of 2 × 10^5^ per well and cultured overnight in RPMI 1640 complete medium. The cells were treated with DOX/HA-b-PCDA NPs or free DOX at a DOX concentration of 5 μg/mL for 72 h. At the end of the treatment, the cells were collected via centrifugation and stained using an Annexin V-FITC/BUV450-A Apoptosis Detection Kit (Bestbio, Shanghai, China). Apoptotic cells were detected using the FACSCalibur device (BD). To assess the apoptotic rate of BCSCs after different treatments, BCSC-enriched 4T1 mammospheres were dissociated into single cells. The dissociated cells were then seeded in ultralow attachment 6-well plates at a density of 2 × 10^5^ per well and cultured in serum-free media for 72 h. The treatments were the same as those used for the 4T1 BCCs.

### Disruptive effects of NPs on already existing BCSC-enriched 4T1 mammospheres

The capacity of NPs to disrupt existing BCSC-enriched 4T1 mammospheres was evaluated to determine its inhibitory effect on the self-renewal capacity of CSCs [53]. Briefly, BCSC-enriched 4T1 mammospheres developed from 40,000 cells per well in ultralow attachment 6-well plates were treated with DOX/HA-b-PCDA NPs or free DOX at a DOX concentration of 5 μg/mL for 7 d. The morphology of BCSC-enriched 4T1 mammospheres was captured via inverted microscopy using an IX81 instrument (Olympus, Tokyo, Japan).

### Effects of NPs on the prevention of secondary BCSC-enriched 4T1 mammosphere formation

The ability of NPs to prevent the formation of secondary BCSC-enriched 4T1 mammospheres was evaluated to further evaluate their inhibitory effect on the self-renewal capacity of CSCs [53]. BCSC-enriched 4T1 mammospheres were dissociated into single cells, which were then seeded in ultra-low attachment 6-well plates at a density of 2 × 10^5^ cells per well and cultured in a serum-free medium. DOX/HA-b-PCDA NPs or free DOX was added to each well at a DOX concentration of 5 μg/mL and incubated with 4T1 mammosphere cells for 7 d. At the end of the treatment scheme, the mammospheres were imaged using a microscope.

### Determination of the proportion of ALDH1^high^ cells after NP treatment in vitro

BCSC-enriched 4T1 mammosphere cells were seeded in ultra-low attachment 24-well plates in serum-free medium. After 24 h, the cells were treated with DOX/HA-b-PCDA NPs, HA-b-PCDA NPs, or free DOX at a DOX concentration of 5 μg/mL for 48 h. At the end of treatment, the cells were stained with Aldefluor fluorescent reagent. ALDH1^high^ cells from each group were analyzed using FACSCalibur flow cytometry system (BD).

### Establishment of a 4T1 tumor-bearing mice model

Female BALB/c mice (5–6 weeks old) were purchased from the Guangdong Experimental Animal Center (Guangzhou, China). 4T1 BCCs (4 × 10^6^ cells in 0.2 mL PBS) were injected subcutaneously into the right axilla of the mice to establish 4T1 tumor.

### In vivo tumor-targeting and biodistribution

When the tumor volume reached approximately 200 mm^3^, Dir/HA-PCDA NPs or free Dir solution was injected into mice via the tail vein at a fixed Dir dose of 0.5 mg/kg. At predetermined time points after injection (0, 1, 2, 4, 8, 24 and 48 h), the treated mice were anesthetized, and fluorescence images were recorded using an IVIS Spectrum in vivo imaging system (PerkinElmer, Waltham, MA, USA). The mice were sacrificed at 48 h after injection, and the major organs (heart, liver, spleen, lung, and kidney) and tumors of the sacrificed mice were harvested. The fluorescence signals in these organs and tumors were measured using the aforementioned imaging system.

### In vivo BCSC accessibility

The in vivo BCSC accessibility of HA-b-PCDA NPs was detected using a 4T1 tumor model. When the tumor volume reached approximately 200 mm^3^, Dir/HA-b-PCDA NPs or free Dir solution was injected into mice via the tail vein at a fixed Dir dose of 0.5 mg/kg. Four hours after injection, the mice were sacrificed, and the tumor tissues were harvested and embedded in the tissue-freezing medium for cryostat sectioning to create 10-μm slices using a CM1950 instrument (Leica, Wetzlar, Germany). The tumor sections were stained with a specific antibody against ALDH1A1, and then incubated with Alexa Fluor-488 labeled goat anti-rabbit IgG (H + L) as the secondary antibody (Beyotime). After staining with DAPI, CLSM was employed to record the location of the NPs via the green fluorescence of the secondary antibody. The accessibility of NPs to BCSCs was depicted as the colocalization of the red fluorescence signals of NPs and the green fluorescence signals of BCSC marker.

### In vivo antitumor efficacy

4T1 tumor-bearing mice with a tumor volume approximately 150 mm^3^ were divided into three drug administration groups (*n* = 6) and one negative control group (*n* = 6). The drug administration groups were respectively treated with free DOX, DOX/HA-b-PCDA NPs, and HA-b-PCDA NPs at a DOX dose of 1 mg/kg and/or HA-b-PCDA NPs dose of 20 mg/kg via intravenous injection every 3 d. The negative control group was treated with normal saline (NS) solution. Tumor growth was monitored by measuring the length and width of the tumor every 2 d and calculating the tumor volume as 1/2 × length × width^2^. The body weights of mice were recorded during treatment. At the end of the treatment, the mice were sacrificed, and the tumor and major organs were harvested. The tumor growth inhibitory (TGI) rates were calculated according to the weight of the tumors as (1-mean tumor weight of the drug treatment group/mean tumor weight of the negative group) × 100%. To further verify the antitumor efficacy of each treatment, the tumor tissues were fixed with 10% neutral buffered formalin overnight. Tumor mass from each group were embedded in paraffin and sectioned at 5 μm for hematoxylin and eosin (H&E) and erminal deoxynucleotidyl transferase dUTP nick-end labeling (TUNEL) analysis. To determine whether HA-b-PCDA could eliminate BCSCs in vivo, the tumor sections were incubated with a specific antibody against ALDH1 followed by an Alexa Fluor 488 labeled gloated anti-rabbit IgG (H + L) secondary antibody. After the nuclei were stained with DAPI, fluorescence microscopy was used to observe fluorescence signals in the tumor sections. In addition, ALDH1 expression in the tumor tissues of different groups after treatment was measured using WB.

### Transcriptome sequencing

After treatment with PBS or 100 μg/mL HA-b-PCDA for 4 d, the two groups (three replicates for each group) of BCSC-enriched 4T1 mammosphere cells were lysed with TRIzol® reagent (Invitrogen, California, USA). RNA-sequencing was then performed by Guangzhou IGE Biotechnology Ltd. (Guangzhou, China) on a HiSeq^TM^2000 platform (Illumina, San Diego, CA, USA). Clean reads were obtained and compared with the reference genome sequence. The number of reads in each gene was counted according to the position of the genome annotation file, and the reads were compared to the genome. Statistical tests were performed to identify differentially expressed genes. Finally, the differentially expressed genes were annotated using function, the Kyoto Encyclopedia of Genes and Genomes pathway (KEGG) tests to determine the enrichment of biological processes and signaling pathways.

### RT-qPCR

After treatmemnt with 0, 20, and 100 μg/mL HA-b-PCDA for 4 d, the BCSC-enriched 4T1 mammosphere cells were collected, and total RNA was isolated with TRIzol® reagent (Invitrogen), and quantified using NanoPhotometer-NP80 (Implen, Munich, Germany). Thereafter, 1 μg total RNA was reverse transcribed into cDNA using the RevertAid (ThermoFisher Scientific, Darmstadt, Germany). RT-qPCR was conducted using Hieff® qPCR SYBR® Green Master Mix (YEASEN, Shanghai, China) on the LightCycler 480 instrument (Roche, Mannheim, Germany). The relative expression levels of target genes were normalized using β-Actin. the fold change was calculated using the 2^−ΔΔct^ method. The primer sequences used for RT-qPCR are listed in Table S4.

### WB

The total proteins in tumor tissues or BCSC-enriched 4T1 mammosphere cells were collected and extracted using ice-cold RIPA lysis buffer (Beyotime) supplemented with protease (Boster, Wuhan, China). and phosphatase (Applygen Technologies, Beijing, China) inhibitors. After denaturation in 5 × SDS-PAGE Sample Loading Buffer (Beyotime, Shanghai, China) at 95 °C for 5–10 min, 30–40 μg protein lysate was separated via 10% SDS-PAGE gel (EpiZyme, Shanghai, China) and transferred onto a PVDF membrane (Merck Millipore, Massachusetts, USA). The membranes were blocked with 5% skim milk for 1–2 h at room temperature, and then incubated with primary antibodies (Table S5) at 4 °C overnight. The membranes were washed three or four times with 1 × Tris-buffered saline Tween (TBST) and incubated with horseradish peroxidase-conjugated secondary antibodies (Table S5) for 1–2 h at room temperature. After washing with TBST buffer, the membranes were visualized using ECL reagent (Millipore, Massachusetts, USA) and photographed with a MiniChemi™ 910 instrument (SAGECREATION, Beijing, China). The grays cale of the bands was evaluated using VisionWorks software (UVP, Upland, CA, USA).

### Statistical analysis

Statistical analysis was performed using an unpaired Student’s t-test (two-tailed) and one-way ANOVA in GraphPad Prism v8.3.0 (GraphPad Software, San Diego, CA, USA). Statistical significance was established at *p* value < 0.05.

## Results

### Synthesis and characterization of HA-b-PCDA

**Fig. 1:**
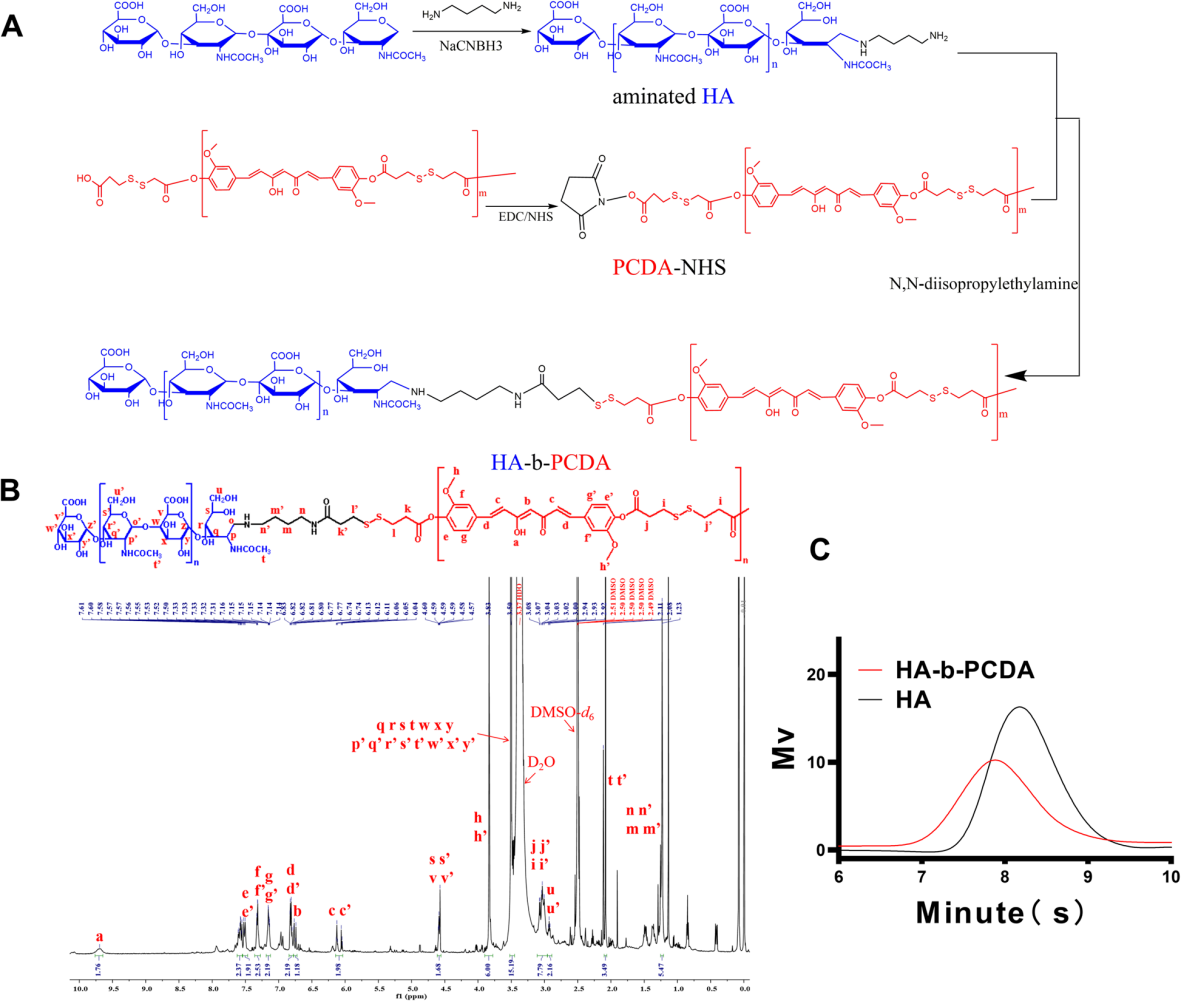
The synthesis and characterization of HA-b-PCDA. **A** The synthetic scheme of HA-b-PCDA. **B** The ^1^H-NNR spectrum of HA-b-PCDA in DMSO-d6. **C** The GPC spectra of HA-b-PCDA and HA

The HA-b-PCDA synthesis scheme is displayed in Fig. 1A. PCDA was polymerized from the phenolic hydroxyl groups of CUR and the carboxyl groups of 3, 3'-dithiodipropionic acid (DA), so the relationship between CUR and DA in PCDA is almost 1:1, this can also be seen from the integration in the ^1^H-nuclear magnetic resonance (^1^H-NMR) of PCDA (Fig. S1). The calculated molecular weight of PCDA is 2923 Da, according to ^1^H-NMR of PCDA. And the the number-average molecular weight (Mn) of PCDA was 3627 Da with a polydispersity index (PDI) of 1.48 based on gel-permeation chromatography (GPC) (Fig. S2 and Table S1). PCDA was synthesized by polymerization of the phenolic hydroxyl group of CUR and the carboxyl group of DA. So, there was another carboxyl group of DA in the end functional group of PCDA (PCDA-COOH). PCDA-COOH was transfered to N-hydroxysuccinimide group of PCDA (PCDA-NHS) and reacted with amino-functionalized HA, forming HA-b-PCDA. In this study, HA with Mn of 7.5 kDa was used in order to make the molecular weight of HA at the hydrophilic end of HA-b-PCDA more than twice that of PCDA at the hydrophobic end. The synthesized HA-b-PCDA was characterized using ^1^H NMR and GPC (Fig. 1B and C, and Table S1). ^1^H NMR spectroscopy confirmed the successful synthesis of the intermediate products of HA-b-PCDA (Fig. S1, S3 and S4). In GPC spectral analysis, the Mn of HA, PCDA and HA-b-PCDA was 8194, 3627, and 10,498 Da, respectively. The Mn of HA-b-PCDA was aligned with the theoretical value (Table S1), indicating the successful synthesis of HA-b-PCDA and the ratio of HA and PCDA in HA-b-PCDA was 1:1.

## HA-b-PCDA as a nanocarrier for chemotherapeutic drug delivery

### Molecular docking and molecular dynamics studies

**Fig. 2:**
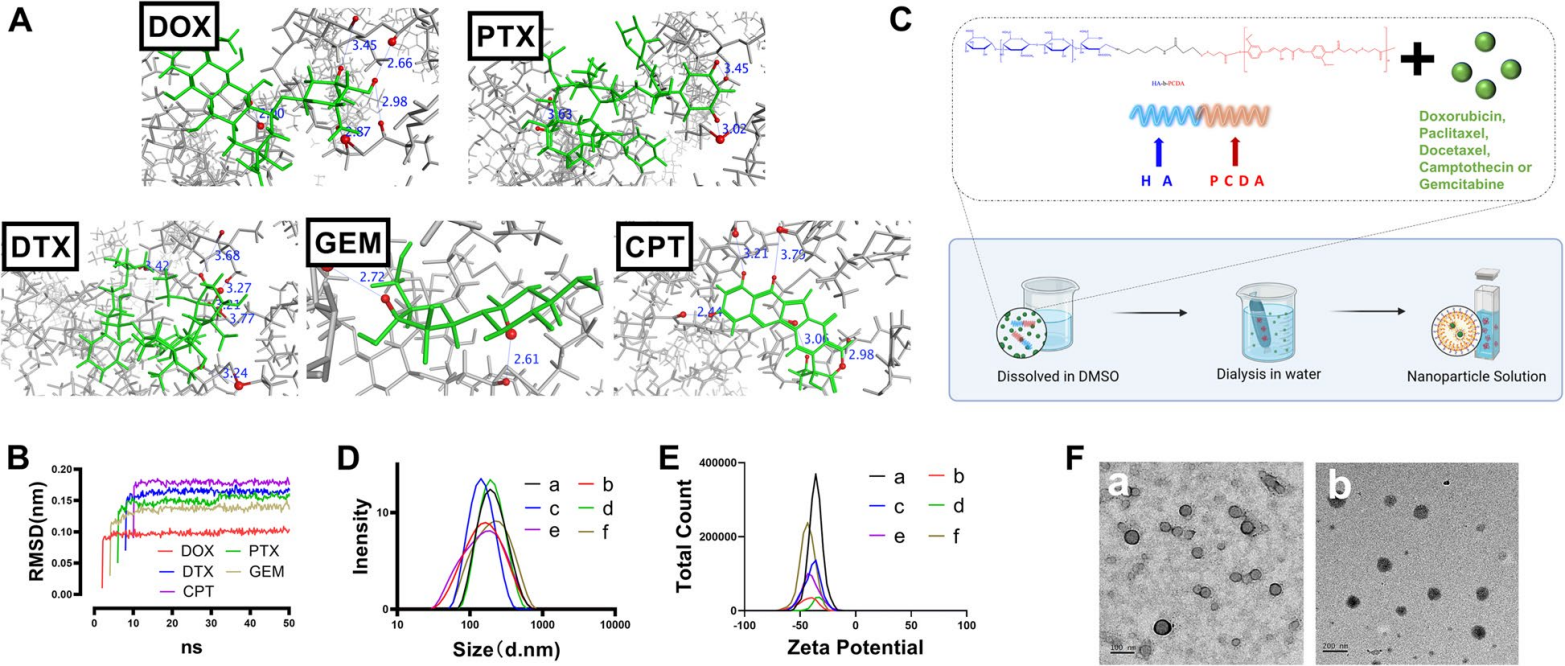
HA-b-PCDA as a nanocarrier to deliver chemotherapeutic drugs. (A) The conformation of the complex consisting of HA-b-PCDA with DOX, PTX, DTX, GEM or CPT obtained by the molecular docking study. (B) RMSD value of the complex of HA-b-PCDA and DOX, HA-b-PCDA and PTX, HA-b-PCDA and DTX, HA-b-PCDA and GEM, or HA-b-PCDA and CPT obtained by the molecular dynamics study. (C) The schematic illustration of the method of HA-b-PCDA to encapsulate chemotherapeutic drugs, forming drug-loaded HA-b-PCDA nanoparticles. (D) The hydrodynamic sizes of DOX/HA-b-PCDA NPs (a), PTX/HA-b-PCDA NPs (b), DTX/HA-b-PCDA NPs (c), GEM/HA-b-PCDA NPs (d), CPT/HA-b-PCDA NPs (e), and HA-b-PCDA NPs (f). (E) The zeta potentials of DOX/HA-b-PCDA NPs (a), PTX/HA-b-PCDA NPs (b), DTX/HA-b-PCDA NPs (c), GEM/HA-b-PCDA NPs (d), CPT/HA-b-PCDA NPs (e) and HA-b-PCDA NPs (f). (F) The morphologies of HA-b-PCDA NPs (a) and DOX/HA-b-PCDA NPs (b) observed by TEM

The 35 anticancer drugs and the constructed HA-b-PCDA binding pose were investigated using AutoDock Vina. HA-b-PCDA hydrophobic residues were employed as affinity grid maps to obtain the conformational affinity energy calculations and interaction binding types with the 35 drugs [54, 55]. AutoDock Vina computed the binding affinity energies of the polymer-drug complexes with binding free energies < -5 kcal/mol, which indicated strong binding of the small molecule chemical drug to HA-b-PCDA [56, 57]. The complex conformations with the lowest docking scores based on AutoDock Vina are shown in Fig. 2A and Fig. S5–S10. Further analysis of these complex conformations revealed the presence of hydrogen bonds and hydrophobic interactions in all bound drugs. Hydrogen-bonding interactions can contribute to the ability of the polymer to loaded drugs. Hydrophobic interaction can increase the binding affinity between the polymer-drug interfaces. As depicted in Table S2, with the exception of testolactone and vinblastine, the other drugs exhibited pi-pi stacking with HA-b-PCDA, which is introduced by the aromatic group and can increase the stability and loading capacity of polymeric micelles. Fiftween drugs displayed cation-π interactions, and sixteen drugs displayed amide stacking in the polymeric complexes. Both amide stacking, such as aryl O–H or N–H aryl interactions, and cation-π interactions could induce closer packing and enhance the pi-pi aromatic interactions. To validate the conformation of molecular docking, a 50 ns long molecular dynamics simulation was performed to demonstrate the stability of the docking pose.

All-atom simulations are necessary for evaluating the dynamics of polymers and understanding their binding to small molecules [58–60]. When RMSD value is lower than 0.2 nm in the simulation, it represents the molecules of polymer-drug complex could stay in a stable stage [59, 61]. Based on the trajectory results, the RMSD was calculated using VMD software to evaluate the degree of variation of the HA-b-PCDA molecule. The RMSD of HA-b-PCDA is between 0.11 and 0.15 nm (Fig. 2B, Fig. S11–S16). Therefore, the complexes of HA-b-PCDA and the small-molecule drugs were stable throughout the molecular dynamics study.

### Preparation and characterization of NPs

The molecular docking and molecular dynamic studies revealed that HA-b-PCDA could encapsulate 35 common clinical chemotherapeutic drugs and form stable complexes. Thus, DOX, PTX, DTX, GEM and CPT were used as model drugs to prepare NPs to further verify the drug delivery ability of HA-b-PCDA. A solvent-dialysis method was applied to prepare the drug-loaded NPs (Fig. 2C). The hydrodynamic diameter, zeta potential, morphology, drug loading capacity and encapsulation efficiency of the prepared NPs were determined. As shown in Fig. 2D-E and Table S3, HA-b-PCDA as a nanocarrier could be used to encapsulate DOX, PTX, DTX, GEM and CPT, forming NPs with hydrodynamic diameters of 120–180 nm and zeta potential of -45-(-30) mv. The drug loading contents were 4.98% for DOX/HA-b-PCDA NPs, 5.45% for HA-b-PCDA NPs, 4.91% for DTX/HA-b-PCDA NPs, 6.28% for GEM/HA-b-PCDA NPs, and 6.31% for CPT/HA-b-PCDA NPs. These drug loading contents were lower that the theoretical drug loading content (9%). Transmission electron microscope (TEM) was used to observe the morphology of the NPs. Both DOX/HA-b-PCDA NPs and HA-b-PCDA NPs were spherical (Fig. 2F). The in vitro drug release behaviors of the drug-loaded NPs were investigated to enable further characterization. As depicted in Fig. S17-S21, biphasic release patterns were observed. In particular, a burst release occurred in the first 24 h, with approximately 30.25% of DOX, 31.21% of PTX, 30.20% of DTX, 35.14% of GEM or 38.64% of CPT were released from the corresponding drug-loaded NPs, followed by sustained release behaviors.

### HA-b-PCDA as a nanocarrier with BCCs and BCSCs targeting ability in vitro

Based on accumulating evidence, mammospheres induced by culturing parent cancer cells in ultralow attachment plates are reliable platforms for enriching CSCs [62, 63]. In this study, BCSC-enriched 4T1 mammospheres were produced using suspension culture of parent 4T1 BCCs. Conventional BCSC markers, ALDH1^high^ and CD44^+^/CD24^−/low^, were used to characterize the stemness of BCSC-enriched 4T1 mammospheres. The proportions of ALDH1^high^ and CD44^+^/CD24^−/low^ cells in parent 4T1 BCCs were quantified as controls. As shown in Fig. S22, BCSC-enriched 4T1 mammospheres were formed after 7 d of culture. The percentages of ALDH1^high^ and CD44^+^/CD24^−/low^ cells in the BCSC-enriched 4T1 mammospheres were 36.8% and 42.3%, respectively, which were 1.76-, and 2.23-fold higher than those of 4T1 BCCs. The collective findings indicate that the BCSC-enriched 4T1 mammospheres generated in this study could be used as a model of BCSCs for further experiments.

**Fig. 3:**
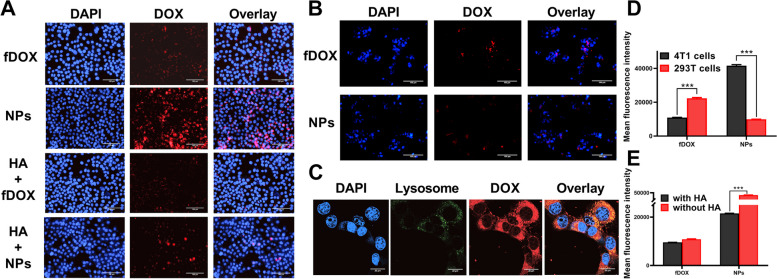
Evaluation of the BCC-targeted capacity of DOX/HA-b-PCDA NPs in vitro. **A**, **B** The uptake of DOX/HA-b-PCDA NPs and free DOX in 4T1 BCCs (**A**) and 293 T cells (**B**) observed by fluorescence microscope, scale bar = 100 μm. **C** Intracellular distribution of DOX/HA-b-PCDA NPs in 4T1 BCCs observed by CLSM, for observation, the nuclei were stained with DAPI (blue), lysosome were stained with lysosome tracker (Green), scale bar = 20 μm. **D** The mean fluorescence intensity of DOX in 4T1 BCCs and 293 T cells after incubation with DOX solution or DOX/HA-b-PCDA NPs solution. **E** The mean fluorescence intensity of DOX in 4T1 BCCs after incubation with DOX solution or DOX/HA-b-PCDA NPs solution with or without pre-incubation with 1 mM of HA solution. “***”, *p* < 0.001

To confirm the selectivity of DOX/HA-b-PCDA NPs for BCCs overexpressing CD44 receptors, their cellular uptake behavior in 4T1 BCCs (overexpressing CD44 receptors) and noncancerous 293 T cells (low-expressing CD44 receptors) was evaluated. As shown in Fig. 3A and B, after treatment with DOX/HA-b-PCDA NPs for 4 h, the intracellular red fluorescence of 4T1 BCCs was stronger than that of 293 T cells. In the DOX-treated group, the intracellular red fluorescence of 4T1 BCCs was weaker than that of 293 T cells. Similar results were obtained using flow cytometry (Fig. 3D; Fig. S23 and S24). Therefore, compared to free DOX, DOX/HA-b-PCDA NPs displayed a higher selectivity for cellular uptake by 4T1 cancer cells than noncancerous 293 T cells. Colocalization experiments verified that DOX/HA-b-PCDA NPs specifically localized to the lysosomes of cells (Fig. 3C), indicating that DOX/HA-b-PCDA NPs entered the cells through endocytosis. To verify the CD44 receptor-targeted cellular uptake of DOX/HA-b-PCDA NPs, a competition assay was performed by pre-incubating the cells with free HA. For the free DOX-treated group, fluorescence intensity did not significantly differ between the cells pretreated with or without excess free HA. In contrast, in the DOX/HA-b-PCDA NP-treated group, the fluorescence intensity of 4T1 BCCs pretreated with excess free HA significantly decreased compared to that without pretreatment with excess free HA (Fig. 3E, Fig. S23).

**Fig. 4:**
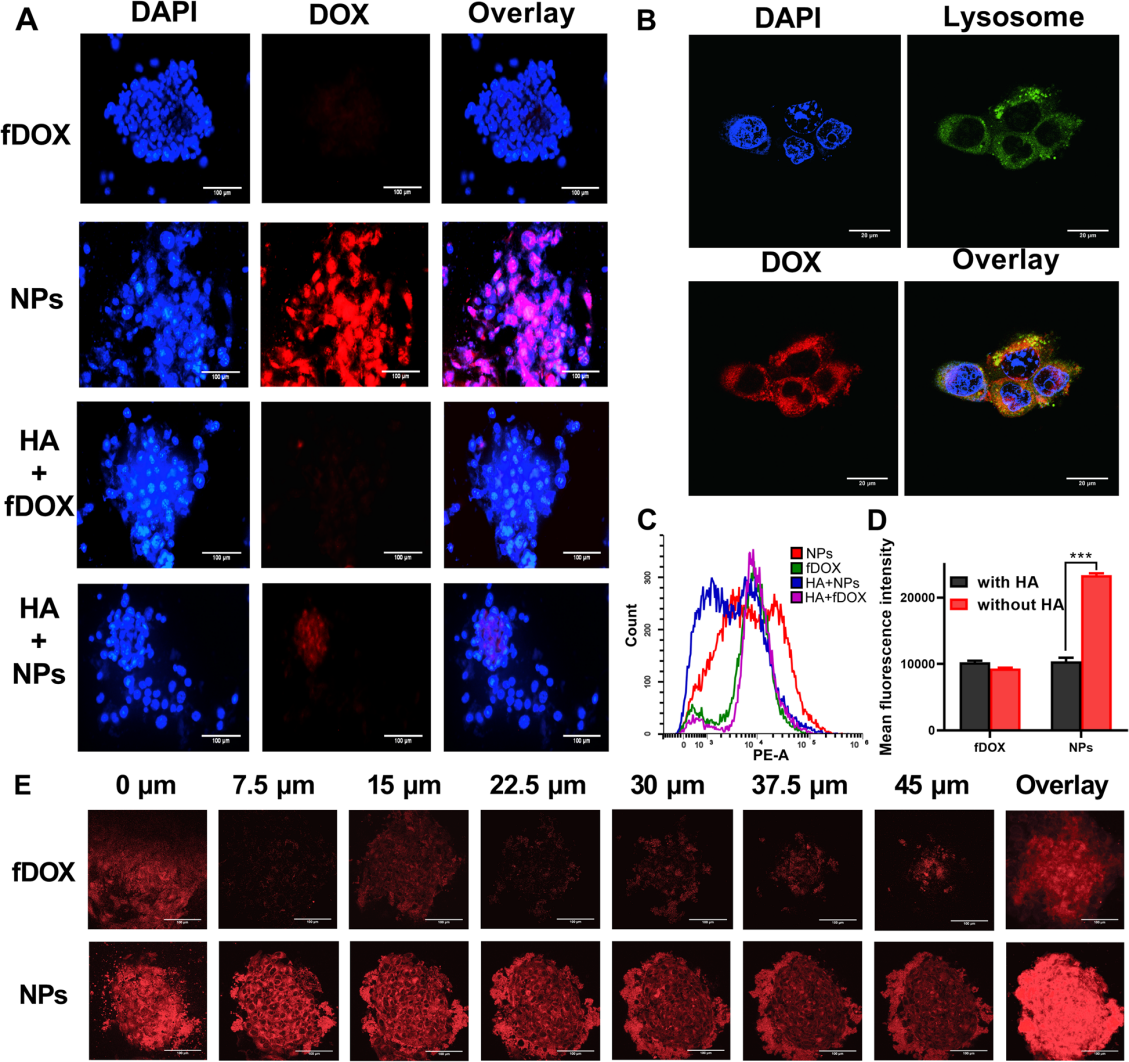
Evaluation of the BCSC-targeted and penetration effects of DOX/HA-b-PCDA NPs. **A** The uptake of DOX/HA-b-PCDA NPs and free DOX by BCSC-enriched 4T1 mammosphere cells under fluorescence microscope, scale bar = 100 μm. **B** Intracellular distribution of DOX/HA-b-PCDA NPs in BCSC-enriched 4T1 mammosphere cells under CLSM, for observation, the nuclei were stained with DAPI (blue), lysosome were stained with lysosome tracker-green. To observe the DOX fluorescence, the excitation wavelength was 553 nm and the detection wavelength was 578–630 nm; to observe the lysotracker-green fluorescence, the excitation wavelength was 493 nm and the detection wavelength was 528–547 nm. scale bar = 20 μm. **C** Typical flow cytometry profiles of the cellular uptake of DOX/HA-b-PCDA NPs and free DOX by BCSC-enriched 4T1 mammosphere cells. **D** The mean fluorescence intensity of DOX in BCSC-enriched 4T1 mammosphere cells after incubation with DOX solution or DOX/HA-b-PCDA NPs solution with or without pre-incubation with 1 mM of HA solution. **E** The DOX fluorescence images observed by CLSM images at different lays of the mammospheres from bottom to top, after incubation with different drug formulation solutions for 4 h, scale bar = 100 μm. “***”, *p* < 0.001

Due to the specific role of BCSCs in tumor initiation, development, and recurrence, an ideal drug delivery system is needed to access BCSCs. Therefore, the BCSC-targeting capacity and intracellular uptake ability of DOX/HA-b-PCDA NPs were evaluated in BCSCs using microscopy and flow cytometry. As depicted in Fig. 4A, stronger red fluorescence signals were observed in these cells after exposure of the BCSC-enriched 4T1 mammosphere cells to DOX/HA-b-PCDA NPs for 4 h. Certain of these signals colocalized with the green signals of lysosomes (Fig. 4B), indicating that the cellular uptake of DOX/HA-b-PCDA NPs by BCSC-enriched 4T1 mammosphere cells occurs through endocytosis. For the free DOX-treated group, the red fluorescence signals were slightly observed in the BCSC-enriched mammosphere cells. The mean fluorescence intensity (MFI) determined using fly cytometry were markedly lower than those of the DOX/HA-b-PCDA NP-treated group. These findings reveal that DOX/HA-b-PCDA NPs enhance the accessibility of DOX to BCSC-enriched 4T1 mammosphere cells compared to free DOX. To further confirm the specific targeting ability of DOX/HA-b-PCDA NPs to BCSC-enriched 4T1 mammosphere cells, competitive binding experiments were performed by pretreating the cells with excess free HA. In the DOX/HA-b-PCDA NP-treated group, when the cells were pre-incubated with free HA to block the CD44 receptors, the red fluorescence signals were obviously decreased (Fig. 4A), and MFI decreased from 23,377.9 to 10,413.8 (Fig. 4C and D). However, in the DOX-treated group, no significant differences were observed in the red fluorescence signals (Fig. 4A) or MFI (Fig. 4C and D) between cells pre-incubated with or without free HA.

To assess the penetration effect of DOX/HA-b-PCDA NPs and free DOX in BCSC-enriched 4T1 mammospheres, the mammosphere was cultured with the drug formulations at 37 °C for 6 h. Thereafter, CLSM was used to investigate the penetration effect. Figure 4E shows the CLSM images captured at different layers of the mammospheres from bottom to top. After incubating the mammospheres with DOX/HA-b-PCDA NPs, strong red fluorescence signals were observed throughout the mammospheres. In contrast, only weak fluorescence was observed at the center of the mammospheres after incubation with free DOX. For example, at the middle layers of 22.5 μm, the red fluorescence intensities after treatment with DOX/HA-b-PCDA NPs were 13-fold stronger than those observed after treatment with free DOX (quantified using ImageJ software). Merged images from different layers of the mammospheres revealed stronger red fluorescence in the inner region of the mammospheres after treatment with DOX/HA-b-PCDA NPs than after treatment with free DOX (Fig. 4E), which may be attributed to the resistance of the mammospheres to free DOX. Three-dimensional (3D) scanned images of BCSC-enriched 4T1 mammospheres also showed that the mammospheres were filled with red fluorescence after culture with DOX/HA-b-PCDA NPs. In contrast, only faint red fluorescence was observed in the mammospheres after culture with free DOX (Fig. S25 and S26).

Flow cytometry was performed to further evaluate the cellular uptake of DOX/HA-b-PCDA NPs and free DOX by ALDH1^high^ or ALDH1^low^ cells in BCSC-enriched 4T1 mammospheres. The results are shown in Fig. S27. After incubation with free DOX, the MFI of DOX in ALDH1^high^ cells was lower than that in ALDH1^low^ cells, indicating that ALDH^high^ cells in BCSC-enriched 4T1 mammospheres were more resistant to free DOX than ALDH^low^ cells. However, no significant differences were observed between the MFI of DOX in ALDH1^high^ and ALDH1^low^ cells after incubation with DOX/HA-b-PCDA NPs. Such finding indicate that DOX/HA-b-PCDA NPs could overcome the resistance to cellular uptake of DOX by ALDH1^high^. After incubation with DOX/HA-b-PCDA NPs, the MFI of DOX in ALDH1^high^, ALDH1^low^ and total cells in the BCSC-enriched 4T1 mammospheres was markedly stronger than that in cells incubated with free DOX. These results indicate that DOX/HA-b-PCDA NPs could significantly enhance the cellular uptake of DOX by both ALDH1^high^ and ALDH1^low^ cells in BCSC-enriched 4T1 mammospheres. Additionally, CLSM was used to observe the localization of ALDH1^high^ within BCSC-enriched 4T1 mammospheres. The results showed that ALDH1 ^high^ cells were distributed in both core and edge of the BCSC-enriched 4T1 mammospheres, and the density of ALDH1 ^high^ cells in the core was slightly stronger than that in the edge (Fig. S28).

### DOX/HA-b-PCDA NPs simultaneously eradicate BCCs and BCSCs in vitro

**Fig. 5:**
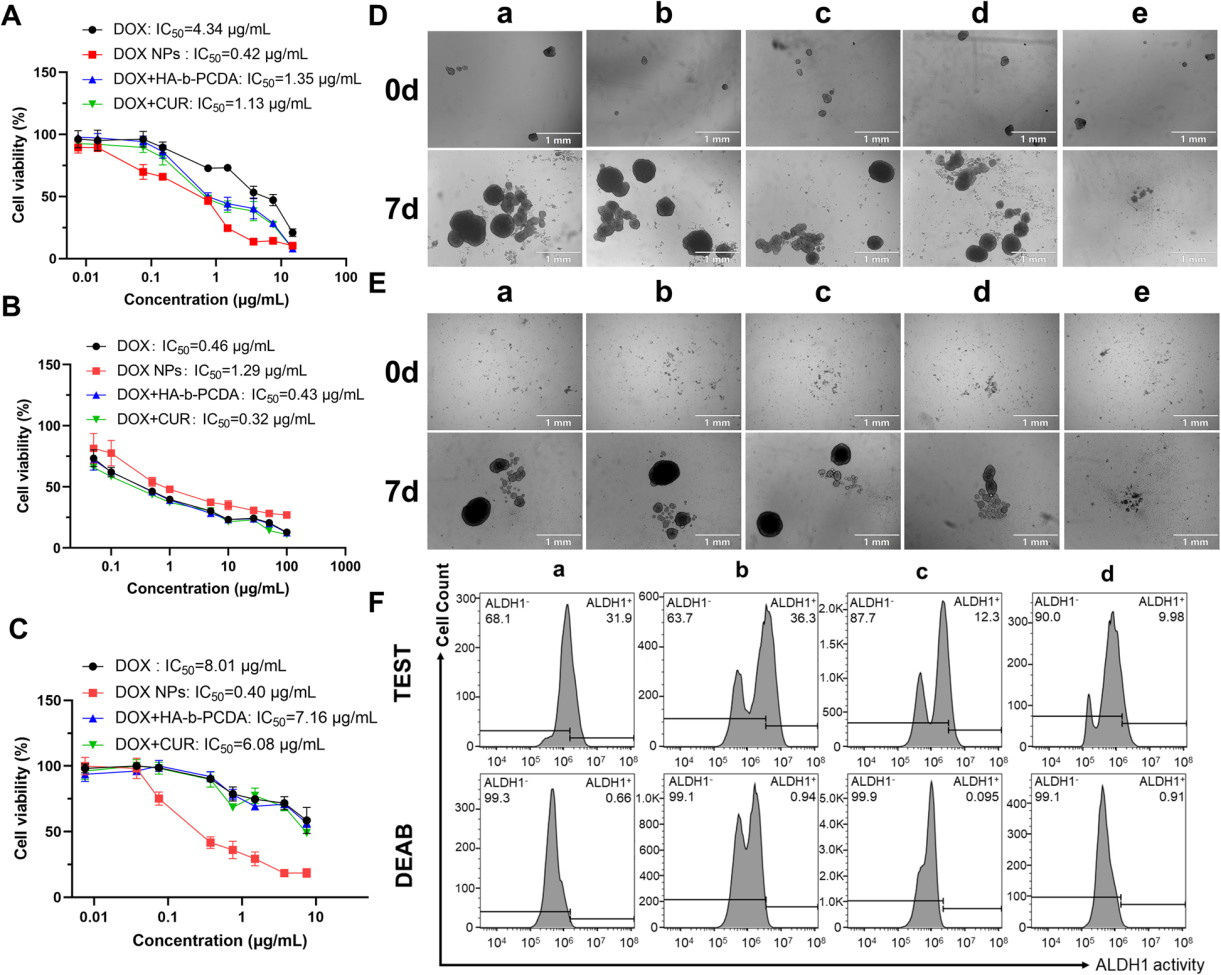
The in vitro anti-BCCs and anti-BCSC efficacy of DOX/HA-b-PCDA NPs. (A-C) The cytotocixity of different drug formulations on the 4T1 BCCs (A) by MTT assay, noncancerous 293T cells by MTT assay (B), and BCSC-enriched 4T1 mammosphere cells by CCK-8 assay (C). (D) The effects of different drug formulations on disrupting already existing BCSC-enriched 4T1 mammosphere. a: control group, b: free DOX treated group, c: free DOX+free CUR treated group, d: free DOX+HA-b-PCDA treated group, e: DOX/HA-b-PCDA NPs treated group, scale bar=1 mm. (E) The effects of different drug formulations on preventing the secondary BCSC-enriched 4T1 mammospheres formation. a: control group, b: free DOX treated group, c: free DOX+free CUR treated group, d: free DOX+HA-b-PCDA treated group, e: DOX/HA-b-PCDA NPs treated group, scale bar=1 mm. (F) The effects of different drug formulations on eradicating of ALDH^high^ cells in BCSC-enriched 4T1 mammospheres. a: control group, b: DOX treated group, c: DOX/HA-b-PCDA NPs treated group, d: HA-b-PCDA NPs treated group

MTT assay was performed to measure the cell inhibitory ability of different drug formulations in 4T1 BCCs and non-cancerous 293 T cells. As shown in Fig. 5A and B, all of the drug formulations inhibited 4T1 and 293 T cell viability in a concentration-dependent manner. Moreover, DOX/HA-b-PCDA NPs displayed stronger cytotoxicity to 4T1 BCCs than in the 293 T cells, whereas free DOX had the opposite effect. The half-maximal inhibitory concentration (IC_50_) values of the different drug formulations were calculated to confirm these findings. The obtained IC_50_ of DOX/HA-b-PCDA NPs against 4T1 BCCs was 0.42 μg/mL, which was 33% of the IC_50_ against 293 T cells. In contrast, the IC_50_ of free DOX against 4T1 BCCs was 9.43-fold higher than that against 293 T cells. By comparing the IC_50_ of DOX/HA-b-PCDA NPs and free DOX against 4T1 BCCs, the IC_50_ of DOX/HA-b-PCDA NPs was 10% of the value for free DOX. These results indicate that DOX/HA-b-PCDA NPs could significantly improve the anti-cancer effect of DOX and reduce its toxicity to normal cells. The in vitro anti-BCSC capacity of the DOX/HA-b-PCDA NPs was evaluated using the CCK-8 assay (Fig. 5C). BCSC-enriched 4T1 mammosphere cells were used as the BCSC model. The inhibitory effects of different drug formulations on this model were evaluated. The IC_50_ of free DOX against BCSC-enriched 4T1 mammosphere cells was significantly higher than that against the 4T1 BCCs, indicating that BCSC-enriched 4T1 mammosphere cells were resistant to free DOX. Comparison the IC_50_ values of different drug formulations revealed that when DOX was loaded into HA-b-PCDA to form DOX/HA-b-PCDA NPs, its inhibitory ability against BCSC-enriched 4T1 mammosphere cells was significantly increased (IC_50_ of DOX/HA-b-PCDA NPs and free DOX, 0.40 vs. 8.01 μg/mL). Compared with free DOX, the enhanced therapeutic effect of DOX/HA-b-PCDA NPs on both 4T1 BCCs and BCSC-enriched 4T1 mammosphere cells could be partly due to the increased cellular uptake of DOX by the cells in the DOX/HA-b-PCDA NP-treated group. Moreover, at the IC_50_ of DOX/HA-b-PCDA NPs against 4T1 BCCs and BCSC-enriched 4T1 mammosphere cells, the corresponding concentrations of the carrier (HA-b-PCDA) were 8.48 and 7.94 μg/mL, respectively, the cell viabilities of 293 T cells after treatment with HA-b-PCDA among the concentrations of 0 to 200 μg/mL were all above 90% (Fig. S29). These findings indicate that HA-b-PCDA is a safe drug carrier in vitro.

To further investigate the cytotoxicity of DOX/HA-b-PCDA NPs against both 4T1 BCCs and BCSC-enriched 4T1 mammosphere cells, apoptosis was evaluatd after different treatments using an Annexin V-FITC/BUV450-A kit (Fig. S30 and S31). The total apoptotic rate of 4T1 BCCs after treatment with DOX/HA-b-PCDA NPs was 29.9%, which was approximately 1.4-fold higher than that obtained after treatment with free DOX and 1.2-fold higher than that of treatmemt with free DOX + free CUR. For BCSC-enriched 4T1 mammosphere cells, the total apoptotic rate of the DOX/HA-b-PCDA NP-treated group was 3.1-fold higher than that of the free DOX-treated group and 1.5-fold higher than that of treatment with free DOX + free CUR. These results indicate that DOX/HA-b-PCDA NPs enhance the capability of DOX to induce apoptosis of both BCCs and BCSCs.

Based on the efficient accessibility of DOX/HA-b-PCDA NPs to BCSC-based 4T1 mammospheres and their excellent cytotoxicity against BCSCs determined using the CCK-8 and Annexin V-FITC/BUV450-A kits, their effects on disrupting existing 4T1 mammospheres were examined. The mammospheres with diameter of approximate 70 μm were selected for the study. After 7 d of treatment, the diameters of most 4T1 mammosphere were larger than 250 μm in the PBS- and free DOX-treated groups; however, the 4T1 mammospheres were significantly destroyed in the DOX/HA-b-PCDA NP-treated group (Fig. 5D). These results indicate that DOX/HA-b-PCDA NPs can eradicate BCSC-enriched mammosphere cells. The ability of DOX/HA-b-PCDA NPs to prevent the formation of secondary 4T1 mammospheres was investigated to further evaluate their inhibitory effects on the self-renewal ability of BCSCs. Single mammosphere cells dissociated from BCSC-enriched 4T1 mammospheres were treated with free DOX, free DOX + free CUR, free DOX + HA-b-PCDA, and DOX/HA-b-PCDA NPs, and then cultured in serum-free media in ultralow attachment plates for 7 d. As shown in Fig. 5E, large mammospheres with diamer larger than 200 μm were observed in the PBS- or free DOX-treated groups. In contrast, only small mammospheres or single cells were observed in the DOX/HA-b-PCDA NP-treated group.

The effect of HA-b-PCDA on reducing the proportion of ALDH^high^ cells in BCSC-enriched 4T1 mammospheres was further investigated. Single mammosphere cells dissociated from BCSC-enriched 4T1 mammospheres were respectively treated with free DOX, HA-b-PCDA, and DOX/HA-b-PCDA NPs and cultured for 48 h. Subsequently, ALDH^high^ cells in each group were analyzed by flow cytometry and fluorescence microscopy. As shown in Fig. 5F, the proportions of ALDH^high^ cells in the HA-b-PCDA NP- and DOX/HA-b-PCDA NP-treated groups were significantly reduced to 9.98% and 12.30% from the 31.9% value of the negative control group. In contrast, the proportion of ALDH^high^ cells was unexpectedly increased in the free DOX-treated group, which may be ascribed to the ability of free DOX to inhibit non-BCSCs with no effect on the BCSCs in 4T1 mammospheres, leading to the enhancement of BCSCs in mammosphere cells. Similar results were obtained by fluorescence microscopy observation (Fig. S32). In addition, as shown in Fig. S32 the nucleus sizes of DOX-treated cells were bigger than other groups. Previous studies have also reported that DOX treatment can induce nuclear swelling and disruption of nucleus membrane structure, as well as nucleus enlargement [64–66]. The mechanisms underlying these nucleus changes are not fully elucidated but may be related to DOX's effects on cell growth and division [67–69].

Besides, compared to the corresponding free drug, corresponding free drug + free CUR, or corresponding free drug + HA-b-PCDA, PTX/HA-b-PCDA NPs, DTX/HA-b-PCDA NPs, GEM/HA-b-PCDA NPs, and CPT/HA-b-PCDA NPs exhibited significantly enhanced the cytotoxicity against BCSC-enriched 4T1 mammosphere cells (Fig. S33–S36) and reduced ALDH1 expression in these cells in vitro (Fig. S37–S40). These results further demonstrate that HA-b-PCDA is a nanocarrier that can eradicate BCSCs and deliver chemotherapeutic drugs in vitro.

### In vivo tumor-targeting efficacy and BCSC accessibility of NPs

**Fig. 6:**
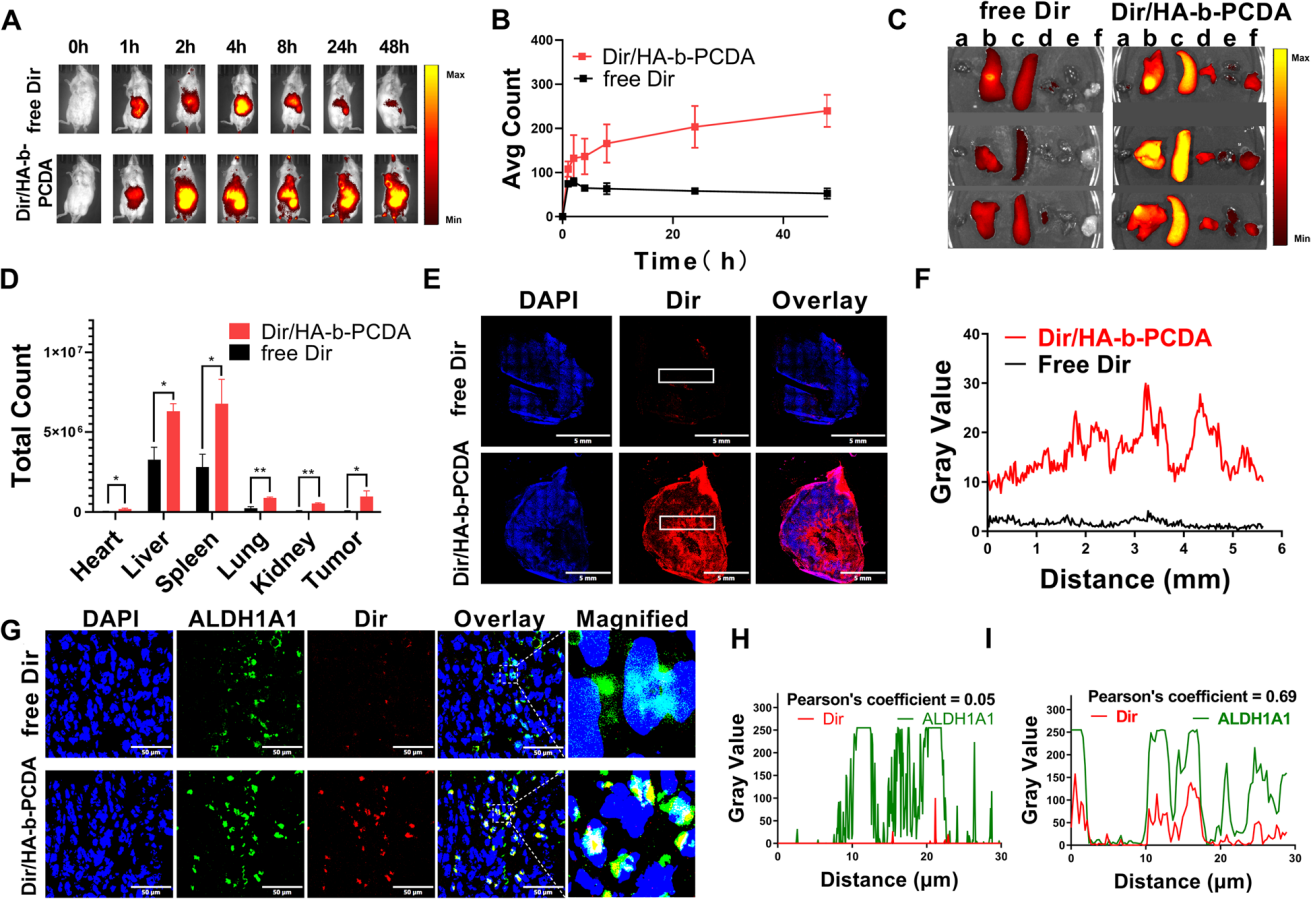
The in vivo tumor-targeted efficacy and BCSC-accessibility of HA-b-PCDA NPs. (A) In vivo fluorescence images of 4T1 tumor-bearing mice at different time points (0, 1, 2, 4, 8, 24 and 48 h) after administration of free Dir or Dir/HA-b-PCDA NPs. (B) Relative radiant fluorescence intensities of tumor areas in (A) at predetermined administration time points, *n*=3. (C) Ex vivo fluorescence images of the tumor and major organs at 48 h post-administration of free Dir or Dir/HA-b-PCDA NPs. a: Heart, b: Liver, c: Spleen, d: Lung, e: Kidney, f: Tumor. (D) Semi-quantification distribution of free Dir or Dir/HA-b-PCDA NPs in tumors and major organs at 48 h post-administration. (E) The permeation of free Dir or Dir/HA-b-PCDA NPs in the whole tumor mass, scale bar=5.0 mm. (F) Imaging analysis of the permeation of free Dir or Dir/HA-b-PCDA NPs by Image-Pro analyzer. (G) CLSM imaging of the access of free Dir or Dir/HA-b-PCDA NPs to the BCSCs in 4T1 tumors, scale bar=50 μm. (H, I) Imaging analysis of the BCSC-accessibility of free Dir (H) or Dir/HA-b-PCDA NPs (I) using Fiji (is just ImageJ) software. “*”, *p*<0.05; “**”, *p*<0.01

To investigate the tumor-targeting efficacy of the HA-b-PCDA NPs, the near-infrared fluorescent dye Dir was loaded into the HA-b-PCDA NPs to form Dir-loaded HA-b-PCDA NPs (Dir/HA-b-PCDA NPs). Dir/HA-b-PCDA NPs and free Dir were injected into 4T1 tumor-bearing mice via the tail vein for imaging. As shown in Fig. 6A and B, in the Dir/HA-b-PCDA NP-treated mice, the fluorescence signal became obvious in tumor site 2 h after the injection and gradually increased up to 48 h. In contrast, in the free Dir-treated mice, the fluorescence signals in the tumor were feeble during the monitored periods. Major organs and tumors excised from mice at 48 h post-administration were imaged, and the captured ex vivo fluorescence images are depicted in Fig. 6C. The fluorescence signal in isolated tumor tissues of Dir/HA-b-PCDA NP-treated mice was 17.7-fold stronger than that of free Dir-treated mice (Fig. 6D). These results demonstrate that Dir/HA-b-PCDA NPs can targete tumors. This capability was mainly due to the passive targeting ability mediated by the EPR effect of NPs and the active targeting mediated by HA and CD44 interactions. Then, the tumor mass was sectioned to measure the intratumor permeation of Dir and Dir/HA-b-PCDA NPs in tumors by recording their fluorescence signals with CLSM. The red fluorescence signals of Dir/HA-b-PCDA NPs were recorded with high intensity in the whole tumor profiles, but only faint red fluorescence signals of free Dir were recorded (Fig. 6E). The better intratumor permeation of Dir/HA-b-PCDA NPs compared with free Dir was also confirmed by image analysis with Image J (Fig. 6F). The notable tumor targeted and permeated efficacy of Dir/HA-b-PCDA NPs may facilitate their access to BCSCs in 4T1 tumors.

To evaluate the accessibility of HA-b-PCDA NPs to BCSCs in 4T1 tumors, BCSCs were labeled with aldehyde dehydrogenase 1 family member A1 (ALDH1A1) using immunofluorescence staining and detected by CLSM. As shown in Fig. 6G, the red fluorescence intensity of Dir in the Dir/HA-b-PCDA NP-treated group was significantly stronger than in the free Dir-treated group. In addition, the colocalization of Dir/HA-b-PCDA NPs with the ALDH1A1-expressing BCSCs fraction in 4T1 tumors was obviously higher than that of free Dir, and the colocalization index of Dir/HA-b-PCDA NPs with ALDH1A1-expressing BCSCs increased 9.12-fold relative to that with free Dir. Image analysis revealed that the fluorescence signals of Dir/HA-b-PCDA NPs largely overlapped with the signals of BCSCs in 4T1 tumors. However, the signals of free Dir and BCSCs in 4T1 tumors barely colocalized (Fig. 6H and I). These results indicate that HA-b-PCDA NPs have in vivo accessibility to the ALDH^high^ BCSC subpopulation in tumors and may have the ability to eliminate BCSCs.

### In vivo antitumor efficacy of DOX/HA-b-PCDA NPs

**Fig. 7:**
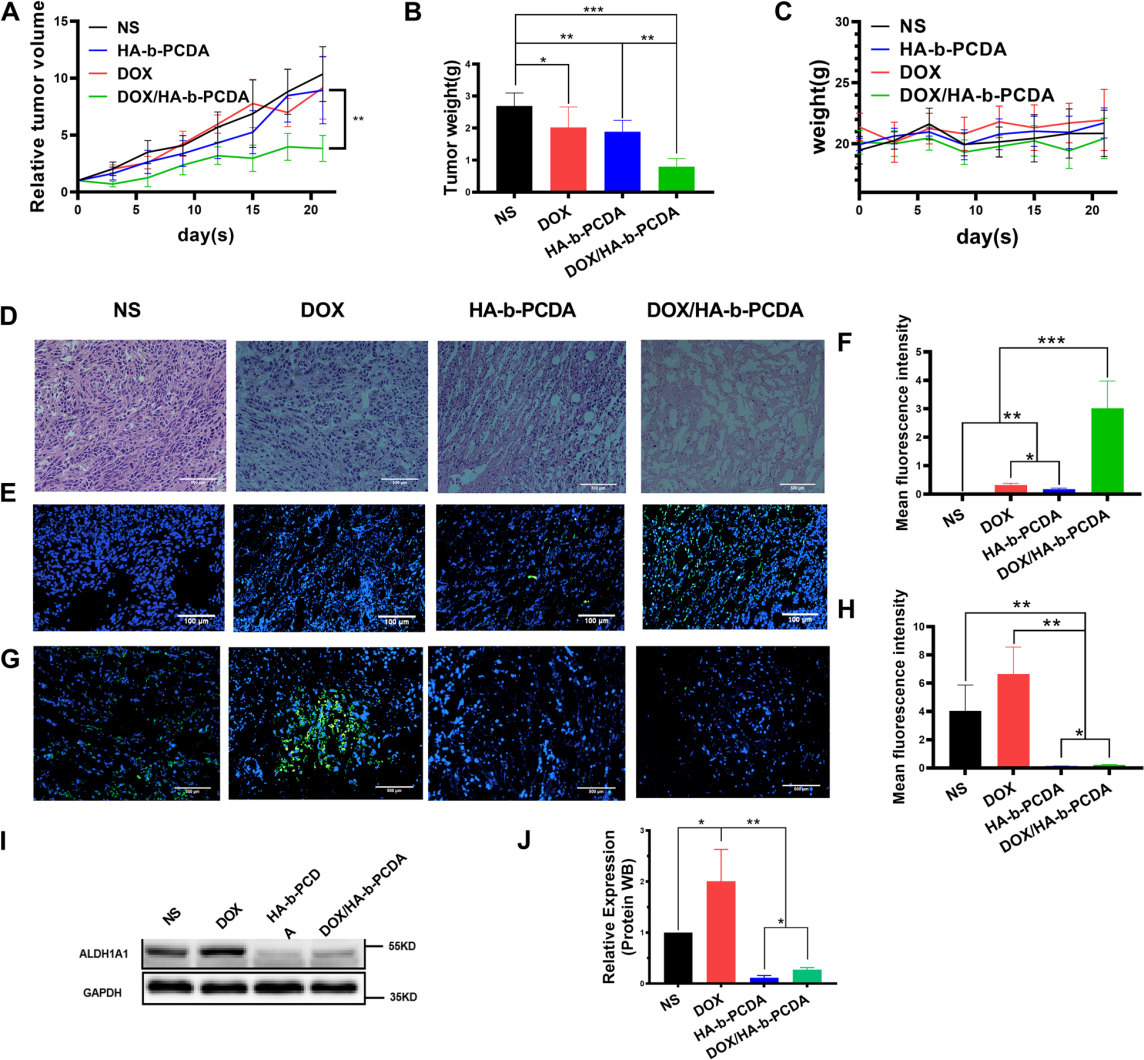
The in vivo antitumor effects of DOX/HA-b-PCDA NPs on 4T1 breast cancer model. **A** The relative tumor volume growth curves from each group. **B** The tumor weight of each group at the end of the treatment. **C** The mice body weight growth curves from each group. **D** Histological analysis of tumor tissue by H&E staining, scale bar = 500 μm. **E** Immunofluorescence staining of apoptosis cells in the tumor tissue at the end of treatment by TUNEL assay, Blue, cell nucleus, Green, apoptosis cell, scale bar = 100 μm. **F** quantified with Fiji (is just ImageJ) software of apoptosis cells in the tumor tissue at the end of treatment by TUNEL assay in (**E**). **G** Immunofluorescent staining of ALDH1^high^ cells in the tumor tissue at the end of the treatment, Blue, cell nucleus; Green, ALDH1, scale bar = 500 μm. **H** Fluorescence intensity of ALDH1.^high^ cells in the tumor tissues (**G**) quantified by Fiji (is just ImageJ) software. **I** Western blotting analysis of the ALDH1 expression. **J** Quantification by densitometry analysis of (**H**). “*”, *p* < 0.05; “**”; *p* < 0.01; “***”, *p* < 0.001

The in vivo antitumor efficacy of the DOX/HA-b-PCDA NPs was investigated using a 4T1-subcutaneous mice model. 4T1-tumor bearing mice with an initial tumor volume of approximately 150 mm^3^ were intravenously injected with NS, free DOX, HA-b-PCDA NPs, or DOX/HA-b-PCDA NPs. As shown in Fig. 7A, at 21 d after treatment with free DOX, and HA-b-PCDA NPs, the tumor volumes were 91.60% and 92% of the negative control group (NS-treated group). The extremely weak inhibition of tumor volume after treatment with free DOX, may be attributed to the low administration dose of DOX (only 1 mg/kg) and the limited tumor-targeting ability of free DOX. In the HA-b-PCDA NP-treated group, although HA-b-PCDA could eradicate CSCs, it had no noticeable inhibitory effect on cancer cells, which may account for its low tumor volume inhibition. In contrast, the tumor volume in DOX/HA-b-PCDA NP-treated mice was obviously smaller than that in mice treated with saline (30% of the negative control group), even though the DOX dose of DOX/HA-b-PCDA NP-treated group was as low as that of the free DOX-treated group. To further verify the enhanced inhibitory ability of DOX/HA-b-PCDA NPs, the tumors were excised and weighed at the end of the experiment. Tumor size was the smallest in the DOX/HA-b-PCDA NPs group (Fig. S41), and the tumor inhibition rate (TIR) calculated from the tumor weight (Fig. 7B) of the treated groups was 74%, 23% and 24% for the groups treated with DOX/HA-b-PCDA NPs, HA-b-PCDA NPs, and free DOX, respectively. Compared with that in the other groups, the higher TIR of the DOX/HA-b-PCDA NP-treated group further demonstrated that DOX/HA-b-PCDA NPs displayed the strongest inhibitory effect on tumor growth among all the treated groups. Of note, the TIR of the DOX/HA-b-PCDA NP-treated group was higher than the sum of the free DOX-treated and HA-b-PCDA NP-treated groups, indicating a synergetic effect of DOX and HA-b-PCDA NPs in prevention of tumor growth. H&E (Fig. 7D) and TUNEL (Fig. 7E and F) staining results further confirmed that the DOX/HA-b-PCDA NP-treated group exhibited the best tumor cell proliferation suppression and apoptosis induction compared to that in the other treatment groups.

To investigate the effects of various treatments on BCSCs, the proportion of ALDH1^high^ cells in the tumor tissue was first determined using immunofluorescence assays and then WB. As shown in Fig. 7G and H, compared to that in the negative control, the fraction of ALDH1^high^ cells in the tumor tissue after free DOX treatment unexpectedly increased, indicating that free DOX treatment resulted in the enrichment of BCSCs in tumors, consistent with the results of other studies [70, 71]. In contrast, the HA-b-PCDA NPs and DOX/HA-b-PCDA NPs led to 97% and 95% reductions in ALDH1^high^ cells in tumor tissue, respectively, demonstrating that the carrier (HA-b-PCDA) exhibited obvious effects on eradicating BCSCs. Similarly, the effect of HA-b-PCDA on reducing the expression of ALDH1 in tumor tissues was also validated using WB (Fig. 7I and J). To preliminarily investigate the effect of DOX/HA-b-PCDA NPs on anti-cancer immunity, we used immunofluorescence to evaluate CD8 expression in tumor tissues. No significant differences in CD8 expression were observed among the groups (Fig. S42). H&E stain of organ obtained from the in vivo antitumor study was used to study the potential organ toxicity after treatments. The results showed that no significantly toxicity of heart, liver, spleen, lung and kidney were observed after treatment with DOX/HA-b-PCDA NPs (Fig. S43). Besides, no remarkable weight loss was observed in mice during the entire treatment period with DOX/HA-b-PCDA NPs (Fig. 7C). Overall, DOX/HA-b-PCDA NPs exhibit excellent antitumor activity with relatively low toxicity in vivo through their anti-BCSC and tumor-targeting effects.

### HA-b-PCDA eradicates BCSCs by activating Hippo and inhibiting the JAK2/STAT3 signaling pathway

**Fig. 8:**
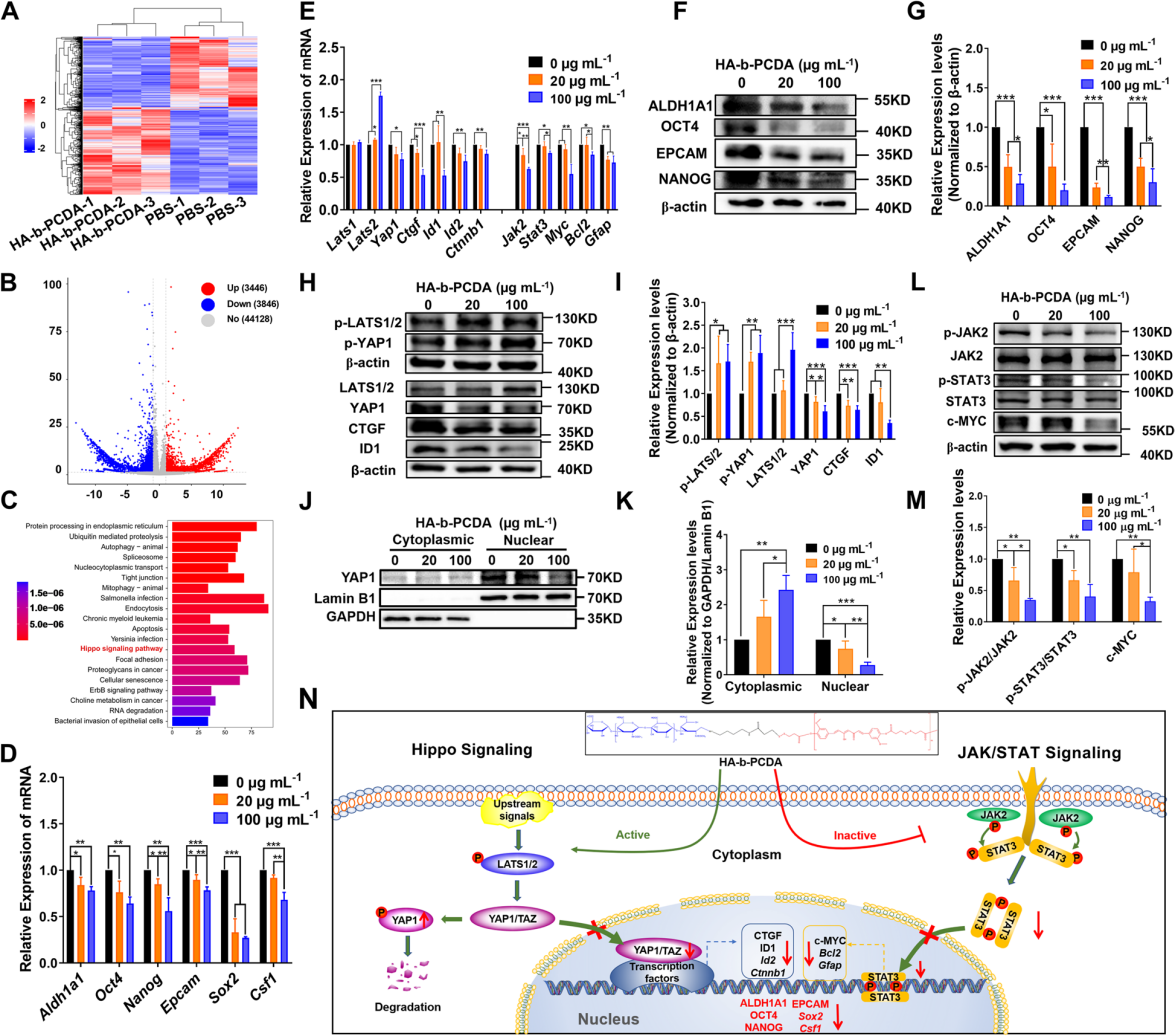
Mechanism of HA-b-PCDA eradicates BCSCs. **A-C** Heatmap (**A**), volcano plot (**B**) and KEGG pathway enrichment analysis (**C**) of DETs of the HA-b-PCDA treated group with negative control groups (*n* = 3). **D** mRNA levels of BCSC markers analyzed by RT-qPCR. **E** mRNA levels of the Hippo and JAK2/STAT3 pathways analyzed by RT-qPCR. **F** Western blot analysis of the protein levels of BCSC markers after treatment with HA-b-PCDA for 4 d. **G** Quantification by densitometry analysis of (**F**). **H** Western blot analysis of protein levels of the Hippo pathway after treatment with HA-b-PCDA for 4 d. **I** Quantification by densitometry analysis of (**H**). **J** The cytosolic and nuclear expression of YAP1 in cells treated with HA-b-PCDA for 4 d. GAPDH and Lamin B1 were used as the loading controls. **K** Quantification by densitometry analysis of (**J**). **L** Western blot analysis of protein levels of the JAK2/STAT3 pathway after treatment with HA-b-PCDA for 4 d. **M** Quantification by densitometry analysis of (**L**). **N** Schematic diagram of the Hippo and JAK2/STAT3 pathways by which HA-b-PCDA eradicates BCSCs. “*”, *p* < 0.05; “**”, *p* < 0.01; “***”, *p* < 0.001

To reveal the underlying molecular mechanism of HA-b-PCDA in the eradication of BCSCs, transcriptome sequencing was first applied to measure the variation in the gene expression of HA-b-PCDA-treated BCSCs compared to cells treated with the negative control. Thereafter, RT-qPCR and WB were performed to further investigate and verify the molecular targets of HA-b-PCDA against BCSCs. Significantly differentially expressed transcripts (DETs) between the HA-b-PCDA-treated and negative control groups were identified and described using heatmaps and volcano plots. After a comparion with the negative control group (|log2 fold-change|≥ 1.0 and p value < 0.05), a total of 7,292 DETs were identified, including 3,446 up-regulated and 3,846 down-regulated transcripts (Fig. 8A and B). The KEGG analysis of the 7,292 DETs was performed using the RNA-seq data. Figure 8C shows the top 20 enriched KEGG pathways. Among these, the Hippo signaling pathway was reported to be closely related to CSCs [72, 73]. Thereafter, the effects of HA-b-PCDA on the Hippo pathway in BCSCs were further verified using RT-qPCR and WB.

RT-qPCR revealed significant changes in *Last2, Yap1, Ctnnb1, Ctgf (Cnn2), Id1, Id2* and *Myc* levels related to the Hippo signaling pathway after HA-b-PCDA treatment (Fig. 8E). WB results indicated increased phosphorylation of LATS1/2 and YAP1, and decreased total YAP1 protein and nuclear translocation of YAP1 proteins after treatment with HA-b-PCDA (Fig. 8H–K). As the HA-b-PCDA concentration increased, the mRNA and protein levels of the three downstream effectors of YAP, CTGF, ID1, and c-MYC were downregulated. In addition to the downstream effectors of YAP, ID1 and c-MYC have been defined as the markers of BCSCs because ID1( +) cells are enriched for self-renewal in tumorsphere [74, 75] and c-MYC drives a stem-like phenotype in cancer [76]. The collective findings indicate that HA-b-PCDA significantly inhibits YAP expression by upregulating LATS1/2 phosphorylation in BCSCs, leading to decreased nuclear translocation of YAP and deactivation of its downstream effectors, which are related to the stemness of BCCs.

As shown in Fig. 8D, HA-b-PCDA downregulated the mRNA expression levels of CSC markers *Aldh1a1, Epcam, Nanog, Oct4, Sox2* and *Csf1*. Furthermore, the suppressive effects of HA-b-PCDA on the protein levels of ALDH1A1, EPCAM, NANOG, and OCT4 were verified (Fig. 8F–G). Besides as a survival factor for breast CSC formation, c-Myc is also a downstream molecule of JAK-STAT signaling pathway [77]. So, the effects of HA-b-PCDA on JAK2/STAT3 signaling pathway of BCSCs was also investigated. HA-b-PCDA downregulated the mRNA expression of *Jak2, Stat3, Myc, Bcl2,* and *Gfap* in 4T1 mammosphere cells (Fig. 8E). Furthermore, the protein expression levels of phosphorylated JAK2 and STAT3, and the ratios of p-JAK2/JAK2 and p-STAT3/STAT3 were suppressed (Fig. 8L and M), indicating the ability of HA-b-PCDA to eliminate BCSCs, partly by inhibiting the JAK2/STAT3 signaling pathway. Overall, the mechanism of HA-b-PCDA eradication of BCSCs may involve the activation of Hippo and inhibition of the JAK2/STAT3 signaling pathway (Fig. 8N).

## Discussion

This study demonstrates the potential of the amphiphilic copolymer, HA-b-PCDA, as an effective nanocarrier for chemotherapeutic drug delivery and eradication of BCSCs. Molecular modeling results verified the ability of HA-b-PCDA to form stable complexes with numerous chemotherapeutic drugs through hydrogen bonding and hydrophobic interactions. DOX/HA-b-PCDA NPs can achieve targeted delivery to BCCs and BCSCs based on the binding of HA and CD44. DOX/HA-b-PCDA NPs increased intracellular DOX levels and penetration into mammospheres compared to free DOX. There are several factors that may contribute to the enhanced penetration of DOX/HA-b-PCDA NPs into BCSC-enriched 4T1 mammospheres [78–83]: (1) mammospheres are enriched in CD44 receptors, which can bind to HA on the surface of NPs; (2) HA modificaition improves the uptake of NPs by cancer cells, as HA is a natural extracellular matrix component that signals tissue repair to cancer cells; (3) HA provides specificity towards cancer cells by exploiting their overexpression of CD44 compared to normal cells. In summary, the HA modification enables DOX/HA-b-PCDA NPs to effectively target and penetrate mammospheres containing BCSCs that are resistant to conventional therapies. In addition to improving DOX efficacy, the HA-b-PCDA carrier itself showed BCSC elimination ability. The dual cytotoxic effects of DOX and HA-b-PCDA led to enhanced apoptosis, disruption of mammospheres, and inhibition of self-renewal. HA-modified nanocarriers could address key limitations of conventional chemotherapy, including poor penetration of solid tumors and inability to target BCSC populations in vitro.

The in vivo studies demonstrated that DOX/HA-b-PCDA NPs can effectively target 4T1 breast tumors in mice through both passive and active effect. EPR effect likely contributed to non-specific accumulation in the tumor site. Additionally, the binding of HA and CD44 provided active targeting to cancer cells overexpressing CD44 receptor. At the tumor tissue level, Dir/HA-b-PCDA NPs showed superior penetration and accessibility to ALDH^high^ BCSCs compared to free Dir. This is a key advantage, as BCSCs play central roles in metastasis, recurrence, and treatment resistance. The HA modification facilitates diffusion into BCSC-enriched mammospheres and interaction with CD44^high^ BCSCs. Combining HA-b-PCDA with DOX chemotherapy yielded DOX/HA-b-PCDA NPs with dual cytotoxic effects against BCCs (from DOX) and BCSCs (from HA-b-PCDA). This resulted in potent inhibition of tumor growth, reduction of BCSC markers, and increased apoptosis compared to free DOX. Several factors likely underlie the performance of DOX/HA-b-PCDA NPs: (1) prolonged circulation and EPR effect enhance tumor accumulation; (2) HA-mediated active targeting increases cancer cell uptake; (3) small size facilitates penetration into tumor tissue; (4) the binding of HA and CD44 enables access to chemoresistant BCSCs; (5) dual cytotoxic effects against both differentiated tumor cells and BCSCs.

Besides, CD8^+^ T cells with anti-tumor functions are the basis of many cancer immunotherapies [84, 85]. So we used immunofluorescence to evaluate CD8 expression in tumor tissues after treatment with NS, DOX, HA-b-PCDA NPs, or DOX/HA-b-PCDA NPs, to preliminarily investigate the potential anti-cancer immunity effect of DOX/HA-b-PCDA NPs. No significant differences in CD8 expression were observed among these groups. This result suggests DOX/HA-b-PCDA NPs may not substantially modulate anti-tumor immune responses, though more in-depth studies are required to fully determine the immunological effects of DOX/HA-b-PCDA NPs. Our future work will further evaluate the anti-cancer immunity effect of DOX/HA-b-PCDA NPs.

Mechanism studies found that HA-b-PCDA eliminated BCSCs by activating Hippo tumor suppressor pathway and inhibiting the JAK/STAT pathway. The Hippo signaling pathway is a tumor-suppressor pathway, The core components of the Hippo signaling pathway include an inhibitory serine/threonine kinase module and a transcriptional module. The first module is composed of sterile 20-like kinases 1/2(MST1/2), large tumor suppressor 1/2 (LAST1/2), and the activating adaptor proteins Salvador homologue 1 (SAV1), MOB kinase activator 1a/1b (MOB1a/1b) [86, 87]. The transcriptional module encompasses the transcriptional co-activator yes-associated protein (YAP) and the transcriptional co-activator with a PDZ-binding motif (TAZ) [88]. When the Hippo signaling pathway is activated (Hippo-ON), the LATS1 and LATS2 cascades cause the phosphorylation of YAP and TAZ, leading to the sequestration and degradation of YAP/TAZ in the cytoplasm. In contrast, when Hippo kinases are inhibited (Hippo-OFF), YAP/TAZ can be transported to the nucleus to bind to transcription factors, such as TEA domain-containing sequence-specific transcription factors (TEAD1-4) and SMAD1-4, ultimately promoting the transcription of target genes that enhance cell proliferation and maintain cell stemness [89]. Emerging evidence indicates that YAP is highly enriched in BCSCs and plays an important role in maintaining stemness [30, 87]. Accordingly, agents that activate the Hippo signaling pathway to inhibit the nuclear transportation of YAP may eradicate BCSCs. Our study demonstrated that HA-b-PCDA increased LATS1/2 kinase activity, leading to YAP phosphorylation, cytoplasmic retention, inhibition of YAP nuclear transcriptional activity, and downregulation of YAP target genes such as CTGF and c-MYC. According to previous research, c-MYC is also a downstream molecule of the JAK/STAT signaling pathway which has important roles in the self-renewal and maintenance of CSCs [77, 90, 91]. Thereafter, we investigated the effects of HA-b-PCDA on the JAK2/STAT3 signaling pathway in BCSCs, and found that HA-b-PCDA could inhibit the JAK2/STAT3 pathway by suppressing the phosphorylated JAK2 and STAT3.

## Conclusion

HA-b-PCDA, capable of targeting BCCs and BCSCs, was successfully synthesized and used to deliver 35 chemotherapeutic drugs. Compared with free DOX, DOX/HA-b-PCDA NPs enhanced the cytotoxicity and apoptosis of BCCs and BCSCs, disrupted BCSC-enriched 4T1 mammospheres, and inhibited the formation and growth of BCSC-enriched 4T1 mammospheres in vitro. Systemic administration of DOX/HA-b-PCDA NPs led to superior antitumor activity in vivo due to their anti-BCSCs, anti-BCCs, and tumor-targeting effects. HA-b-PCDA eradicates BCSCs by activating Hippo and inhibiting the JAK2/STAT3 signaling pathway. Thus, HA-b-PCDA is a nanocarrier that can suppress the stemness of BCCs and potentially deliver numerous clinical chemotherapeutic drugs for effective chemotherapy.

### Supplementary Information

**Additional file 1.** The Synthesis of PCDA, PCDA-NHS, amino-functionalized HA, and HA-b-PCDA were provide in Supplementary Methods. The methods of HA-b-PCDA characterization were also provide in Supplementary Methods. Figure S1−S43 showed ^1^H-NNR and GPC spectrum results, the molecular docking study results, the molecular dynamics study results, cumulative release of drug from drug-loaded HA-b-PCDA NPs in PBS, characterization of the BCSC-enriched mammosphere model, typical flow cytometry profiles of the cellular uptake results, 3D scanned DOX fluorescence images of BCSC-enriched 4T1 mammospheres, evaluation the cellular uptake of DOX/HA-b-PCDA NPs by BCSC-enriched 4T1 mammospheres, the expression of ALDH1 marker in BCSC-enriched 4T1 mammospheres, the in vitro cytotoxiciy of HA-b-PCDA against 293T cells, the results of cell apoptosis study, the expression of ALDH1 marker in BCSC-enriched 4T1 mammoshpere cells after treatment with different drug formulation, the in vitro cytotoxicity of different drug formulations, the expression of ALDH1 marker in BCSC-enriched 4T1 mammoshpere cells after treatments of different PTX formulations, tumor images from different treatment groups, immunofluorescent staining of CD8^+^ cells in the tumor tissue at the end of the treatment, and images of H&E staining of major tissues at the end of different treatment groups. Table S1-S5 showed molecular weight data of intermediate and final products of HA-b-PCDA, the binding energy and interaction type of HA-b-PCDA with related drugs, characterization of the prepared nanoparticles, list of RT-qPCR primers, and antibodies.

## Data Availability

The datasets used and/or analyzed during the current study are available from the corresponding author on reasonable request.

## Abbreviations

BCSCs: Breast cancer stem cells
HA-b-PCDA: Hyaluronic acid-block-poly (curcumin-dithiodipropionic acid)
NPs: Nanoparticles
EPR: Enhanced permeability and retention
CD44: Cluster of differentiation 44
ALDH1: Aldehyde dehydrogenase 1
CSCs: Cancer stem cells
BCCs: Breast cancer cells
DA: 3, 3'-Dithiodipropionic acid
DOX: Doxorubicin
CUR: Curcumin
PTX: Paclitaxel
DTX: Docetaxel
GEM: Gemcitabine
CPT: Camptothecin
RT-qPCR: Quantitative real-time polymerase chain reaction
WB: Western blot
RMS: Root-mean-square
RMSD: Root-mean-square Deviation
VMD: Visual Molecular Dynamics
^1^H NMR: ^1^H-nuclear magnetic resonance
TEM: Transmission electron microscope
LC–MS/MS: Liquid chromatography tandem mass spectrometry
GPC: Gel-permeation chromatography
DMEM: Dulbecco's modified eagle's medium
RPMI: Roswell park memorial institute
TGI: Tumor growth inhibitory
H&E: Hematoxylin and eosin
IC_50_: Half-maximal inhibitory concentration
TUNEL: Terminal deoxynucleotidyl transferase dUTP nick-end labeling
CLSM: Confocal laser scanning microscope
DETs: Differentially expressed transcripts
KEGG: Kyoto Encyclopedia of Genes and Genomes
