# Supplementary material for: A polymeric nanocarrier that eradicates breast cancer stem cells and delivers chemotherapeutic drugs
Source: Biomater Res. 2023 Dec 15;27:133. doi: 10.1186/s40824-023-00465-9 (PMC10722842; doi:10.1186/s40824-023-00465-9)
Supplement: Supplementary file 1 — Additional file 1. The Synthesis of PCDA, PCDA-NHS, amino-functionalized HA, and HA-b-PCDA were provide in Supplementary Methods. The methods of HA-b-PCDA characterization were also provide in Supplementary Methods. Figure S1−S43 showed 1H-NNR and GPC spectrum results, the molecular docking study results, the molecular dynamics study results, cumulative release of drug from drug-loaded HA-b-PCDA NPs in PBS, characterization of the BCSC-enriched mammosphere model, typical flow cytometry profiles of the cellular uptake results, 3D scanned DOX fluorescence images of BCSC-enriched 4T1 mammospheres, evaluation the cellular uptake of DOX/HA-b-PCDA NPs by BCSC-enriched 4T1 mammospheres, the expression of ALDH1 marker in BCSC-enriched 4T1 mammospheres, the in vitro cytotoxiciy of HA-b-PCDA against 293T cells, the results of cell apoptosis study, the expression of ALDH1 marker in BCSC-enriched 4T1 mammoshpere cells after treatment with different drug formulation, the in vitro cytotoxicity of different drug formulations, the expression of ALDH1 marker in BCSC-enriched 4T1 mammoshpere cells after treatments of different PTX formulations, tumor images from different treatment groups, immunofluorescent staining of CD8+ cells in the tumor tissue at the end of the treatment, and images of H&E staining of major tissues at the end of different treatment groups. Table S1-S5 showed molecular weight data of intermediate and final products of HA-b-PCDA, the binding energy and interaction type of HA-b-PCDA with related drugs, characterization of the prepared nanoparticles, list of RT-qPCR primers, and antibodies. [file 40824_2023_465_MOESM1_ESM.docx]

**Supplemental information for**

A polymeric nanocarrier that eradicates breast cancer stem cells and delivers chemotherapeutic drugs

Li Lv^1†^, Yonghui Shi^1†^, Zhicheng Deng^2†^, Jiajia Xu^3†^, Zicong Ye^1^, Jianxiong He^1^, Guanghui Chen^1^, Xiaoxia Yu^1^, Junyan Wu^1*^, Xingzhen Huang^3*^, Guocheng Li^1,2*^

1. Department of Pharmacy, Guangdong Provincial Key Laboratory of Malignant Tumor Epigenetics and Gene Regulation, Sun Yat-Sen Memorial Hospital, Sun Yat-Sen University, Guangzhou 510120, Guangdong, China

2. Shenshan Medical Center, Memorial Hospital of Sun Yat-Sen University, Shanwei 516600, Guangdong, China.

3. School of Pharmacy, Guangxi Medical University, Nanning 530021, Guangxi, China.

^†^ These authors contributed equally to this work.

*Corresponding author

Junyan Wu

[wujunyan@mail.sysu.edu.cn](mailto:wujunyan@mail.sysu.edu.cn)

Xingzhen Huang

[huangxingzhen@gxmu.edu.cn](mailto:huangxingzhen@gxmu.edu.cn)

Guocheng Li

[liguoch@mail.sysu.edu.cn](mailto:liguoch@mail.sysu.edu.cn)

**1. Supplementary materials**

Curcumin was purchased from Chengdu Cologne Chemicals Co., Ltd. 3, 3'-dithiodipropionic acid, dicyclohexylcarbodiimide, and 4-dimethylaminopyridine were purchased from Chengdu Aikda Chemical Reagent Co., Ltd. Anhydrous ether, dichloromethane, N-hydroxysuccinimide, and 1-ethyl-3-(3-dimethylaminopropyl)-carbodiimide hydrochloride were obtained from Chengdu Cologne Chemicals Co., Ltd.

Hyaluronic acid (Mn of 7.5 kDa), 1,4-diaminobutane, Sodium cyanoborohydride and N, N-diisopropylethylamine were purchased Aladdin Reagent Company. Polyethylene glycol poly (methyl methacrylate) standards for GPC were obtained Agilent Technologies Inc.

**2. Supplementary Methods**

**2.1 Synthesis of PCDA**

PCDA was synthesized using our previously reported method with some modifications [1]. Briefly, curcumin (1.02 g), 3, 3'-dithiodipropionic acid (0.562 g), dicyclohexylcarbodiimide (1.009 g) and 4-dimethylaminopyridine (0.121 g) were dissolved in anhydrous dichloromethane (40 mL). The resulting solution was then stirred at room temperature for 24 h. After filtration, anhydrous ether (600 mL) was added to obtain a precipitate. The precipitate was further treated with multiple precipites from dichloromethane and anhydrous ether to purify the product. PCDA (1.038g) was obtained as an orange-yellow powder. ^1^H-NMR（600 MHz，DMSO-d_6_, δ 9.72 (s，1H，**H**OCCHCO-), 7.63 (m，2H， -CC**H**CH-), 7.57 (m，2H，-CCHC**H**C-), 7.52 (m，2H，-CCC**H**C-), 6.98 (m，1H，Ar-C**H**), 6.83 (m，1H，Ar-C**H**), 6.73 (m，1H，OHC=C**H**-CO-), 6.18 (m，1H，Ar-CH=C**H**), 6.12 (m，1H，Ar-CH=C**H**), 3.83 (s，6H，-OC**H**_3_), 3.08 (d，4H，-C**H**_2_CH_2_S**-**), 3.04 (d，4H，-CH_2_C**H_2_**S**-**). The Wn of PCDA was 3627 Da with a polydispersity index (PDI) of 1.48 based on GPC. The ^1^H NMR and GPC spectra of PCDA are depicted in Figure S1 and S2, respectively.

**2.2 Synthesis of PCDA-NHS**

PCDA (0.504 g), N-hydroxysuccinimide (0.1349 g), and 1-ethyl-3-(3-dimethylaminopropyl)-carbodiimide hydrochloride (0.2349 g) were dissolved in anhydrous dichloromethane (40 mL). The mixture was then stirred at room temperature for 20 h. After stirring, the product was precipitated by adding anhydrous ether (40 mL). The precipitate was formed in the refrigerator at 4 °C for 12 h, and dried under vacuum until a constant weight was obtained. PCDA-NHS (0.341g) was obtained as a yellow powder. ^1^H-NMR（600 MHz，DMSO-d_6_）, δ 9.98 (s，1H，**H**OCCHCO-), 7.52 (m，2H， -CC**H**CH-), 7.33 (m，2H，-CCHC**H**C-), 7.15 (m，2H，-CCC**H**C-), 6.99 (m，2H，Ar-C**H**), 6.81 (m，1H，OHC=C**H**-CO-), 6.10 (m，1H，Ar-CH=C**H**), 6.02 (m，1H，Ar-CH=C**H**), 3.83 (s，6H，-OC**H**_3_), 3.05 (m，8H，-C**H**_2_C**H**_2_S**-**), 2.99 (m，4H，-COC**H**_2_C**H**_2_CO**-**), 2.73 (m，8H，-C**H**_2_C**H**_2_S**-**). The ^1^H NMR spectrum of PCDA-NHS is shown in Figure S3.

**2.3 Synthesis of** **amino-functionalized HA**

Amino-functionalized HA was synthesized as previously reported [2]. In brief, HA (1.096 g) was dissolved in an acetate buffer (130 mL). After the complete dissolution of HA, 1,4-diaminobutane (1.0 mL) was added to the HA solution and stirred at 50 °C for 24 h. Sodium cyanoborohydride (0.2 g) was then added to the solution daily for three days. The mixture was purified via dialysis in water for 72 h. At the end of dialysis, the final product was collected and lyophilized, yielding amino-functionalized HA (0.972g) as a white powder. ^1^H-NMR（600 MHz，D_2_O）δ 4.53 (d，2H，-OCC**H**O-), 4.44 (d，2H，-C**H**COOH-), 3.89 (m，2H，-C**H**NHCOCH_3_-), 3.82 (m，2H，-C**H**CH_2_OH-), 3.69 (m，8H，-C**H**OH-), 3.56 (m，3H，-C**H**-), 3.32 (t，2H，-C**H_2_**NH-), 3.00 (m，6H，-C**H**_2_OH, -C**H**_2_NH_2_), 2.00 (s，6H，-COC**H**_3_), 1.71 (m，6H，-C**H**_2_C**H**_2_C**H**_2_**-**). The ^1^H NMR spectrum of the amino-functionalized HA is shown in Figure S4.

**2.4 Synthesis of HA-b-PCDA**

PCDA-NHS (0.280 g), amino-functionalized hyaluronic acid (0.361 g) and N, N-diisopropylethylamine (20 μL) were added to anhydrous dimethyl sulfoxide (20 mL). The resulting mixture was stirred at 50 °C for 48 h. After purification via dialysis in water for 72 h, the final product (HA-b-PCDA) was collected and lyophilized. HA-b-PCDA (0.286 g) was obtained as a yellow powder. ^1^H-NMR（600 MHz，DMSO-d_6_）δ 9.69 (s，1H，**H**OCCHCO-), 7.50 (m，2H， -CC**H**CH-), 7.32 (m，2H，-CCHC**H**C-), 7.15 (m，2H，-CCC**H**C-), 6.97 (m，2H，Ar-C**H**), 6.75 (m，1H，OHC=C**H**-CO-), 6.12 (m，1H，Ar-CH=C**H**), 6.05 (m，1H，Ar-CH=C**H**), 4.59 (m，4H，-C**H**COOH-，-C**H**CH_2_OH-), 3.83 (s，6H，-OC**H**_3_), 3.50 (s，15H，-C**H**NHCOCH_3_-，-C**H**CH_2_OH-，-C**H**OH-，-C**H**-), 3.03 (m，8H，-C**H**_2_C**H**_2_S**-**), 2.93 (m，4H，-C**H**_2_OH), 2.08 (s，6H，-NHCOC**H**_3_), 1.23 (s，8H，-NH(C**H**_2_)_4_NH**-**).The ^1^H NMR, and GPC spectra of HA-b-PCDA are depicted in Figure 2B and 2C, respectively.

**2.5 Characterization of HA-b-PCDA**

The chemical structure and molecular weight of HA-b-PCDA was confirmed using ^1^H-NMR and GPC, respectively. Briefly, after HA-b-PCDA was dissolved in DMSO-d6, its chemical structure was determined using an NMR spectrometer (Avane II 600 MHz, Bruker, Switzerland). After HA-b-PCDA or HA was dissolved in ultrapure water and PCDA-NHS was dissolved in dimethylformamide, their molecular weight was determined using GPC—model 1515 device (Waters, Milford, MA, USA). For GPC analysis of HA and HA-b-PCDA, polyethylene glycol standards with molecular weights ranging from 100 to 1,214,000 Da were used to as calibration standards. For analysis of PCDA, poly (methyl methacrylate) standards with molecular weights ranging from 550 to 1,568,000 Da were used as calibration standards.

**3. Supplementary Figures**

**Figure S1.** The ^1^H-NNR spectrum of PCDA in DMSO-d6

**Figure S2.** The GPC spectrum of PCDA

**Figure S3.** The ^1^H-NNR spectrum of PCDA-NHS in DMSO-d6

**Figure S4.** The ^1^H-NNR spectrum of amino-functionalized HA in D_2_O

**Figure S5.** The conformation of the complex consisting of HA-b-PCDA with sorafenib, imatinib, irinotecan, etoposide or lapatinib obtained by molecular docking study.

**Figure S6.** The conformation of the complex consisting of HA-b-PCDA with nilotinib, carfilzomib, enzalutamide, formestane or elacridar obtained by molecular docking study

**Figure S7.** The conformation of the complex consisting of HA-b-PCDA with edotecarin, exemestane, bexarotene, trametinib or vatalanib obtained by molecular docking study

**Figure S8.** The conformation of the complex consisting of HA-b-PCDA with eribulin, testolactone, vindesine, ixabepilone or erlotinib obtained by molecular docking study

**Figure S9.** The conformation of the complex consisting of HA-b-PCDA with trabectedin, epirubicin, homoharringtonine, vinorelbine or vinblastine obtained by molecular docking study

**Figure S10.** The conformation of the complex consisting of HA-b-PCDA with tretinoin, bosutinib, capecitabine, gefitinib or tamoxifen obtained by molecular docking study

**Figure S11.** RMSD value of the complex of HA-b-PCDA with imatinib, irinotecan, etoposide, lapatinib, or sorafenib obtained by the molecular dynamics study.

**Figure S12.** RMSD value of the complex of HA-b-PCDA with carfilzomib, enzalutamide, formestane, elacridar, or nilotinib obtained by the molecular dynamics study.

**Figure S13.** RMSD value of the complex of HA-b-PCDA with exemestane, bexarotene, trametinib, vatalanib, or edotecarin obtained by the molecular dynamics study.

**Figure S14.** RMSD value of the complex of HA-b-PCDA with testolactone, vindesine, ixabepilone, erlotinib, or erbulin obtained by the molecular dynamics study.

**Figure S15.** RMSD value of the complex of HA-b-PCDA with epirubicin, homoharringtonine, vinorelbine, vinblastine, or trabectedin obtained by the molecular dynamics study.

**Figure S16.** RMSD value of the complex of HA-b-PCDA with bosutinib, capecitabine, gefitinib, tamoxifen, or tretinoin obtained by the molecular dynamics study.

**Figure S17.** Cumulative release of DOX from DOX/HA-b-PCDA NPs in PBS (pH7.4).

**Figure S18.** Cumulative release of PTX from PTX/HA-b-PCDA NPs in PBS (pH7.4).

**Figure S19.** Cumulative release of DTX from DTX/HA-b-PCDA NPs in PBS (pH7.4).

**Figure S20.** Cumulative release of GEM from GEM/HA-b-PCDA NPs in PBS (pH7.4).

**Figure S21.** Cumulative release of CPT from CPT/HA-b-PCDA NPs in PBS (pH7.4).

**Figure S22.** Characterization of the BCSC-enriched mammosphere model. (A) The morphology of 4T1 BCCs, scale bar=100 μm. (B) The morphology of BCSC-enriched mammospheres, scale bar=100 μm. (C) The proportion of CD44^+^/CD24^-/low^ cell in 4T1 BCCs (20.9%). (D) The proportion of CD44^+^/CD24^-/low^ in BCSC-enriched mammospheres(36.8%). (E) The proportion of ALDH1^high^ cell in 4T1 BCCs (18.9%). (F) The proportion of ALDH1^high^ cell in BCSC-enriched mammospheres (42.3%).

**Figure S23.** Typical flow cytometry profiles of the cellular uptake of DOX/HA-b-PCDA NPs and free DOX by 4T1 BCCs with or without the pre-incubation of free HA.

**Figure S24.** Typical flow cytometry profiles of the cellular uptake of DOX/HA-b-PCDA NPs and free DOX by 293T cells.

**Figure S25.** 3D scanned DOX fluorescence images of BCSC-enriched 4T1 mammospheres after incubation with free DOX. Red:DOX; blue:DAPI

**Figure S26.** 3D scanned DOX fluorescence images of BCSC-enriched 4T1 mammospheres after incubation with DOX/HA-b-PCDA NPs. Red:DOX; blue:DAPI

**Figure S27.** Evaluation the cellular uptake of DOX/HA-b-PCDA NPs by BCSC-enriched 4T1 mammospheres. (A-C) Typical flow cytometry profiles show the cellular uptake of DOX/HA-b-PCDA NPs and free DOX by ALDH1^high^ cells (A), ALDH1^low^ cells (B) and total cells (C) in BCSC-enriched 4T1 mammospheres. (D) The mean fluorescence intensity of DOX in 4T1 mammospheres after incubation with DOX solution or DOX/HA-b-PCDA NPs solution. “**”, p<0.01; “***”, p<0.001.

**Figure S28.** The expression of ALDH1 marker in BCSC-enriched 4T1 mammospheres.(A) DAPI; (B) ALDH1; (C) Overlay. scale bar=100 μm.

**Figure S29.** The in vitro cytotoxiciy of HA-b-PCDA against 293T cells

**Figure S30.** The apoptosis of 4T1 BCCs after treatment with different drug formulations analyzed by flow cytometry. (A) PBS treated group; (B) free DOX treated group; (C) free DOX+free CUR treated group; (D) free DOX+HA-b-PCDA treated group; (E) DOX/HA-b-PCDA NPs treated group.

**Figure S31.** The apoptosis of BCSCs-enriched 4T1 mammosphere cells after treatment with different drug formulations analyzed by flow cytometry. (A) PBS treated group; (B) free DOX treated group; (C) free DOX+free CUR treated group; (D) free DOX+HA-b-PCDA treated group; (E) DOX/HA-b-PCDA NPs treated group.

**Figure S32.** The expression of ALDH1^high^ markers in BCSC-enriched 4T1 mammosphere cells after different treatments, observed by fluorescence microscope, scale bar=100 μm.

**Figure S33.** The in vitro cytotoxicity of different PTX formulations against BCSC-enriched 4T1 mammosphere cells.

**Figure S34.** The in vitro cytotoxicity of different DTX formulations against BCSC-enriched 4T1 mammosphere cells.

**Figure S35** The in vitro cytotoxicity of different GEM formulations against BCSC-enriched 4T1 mammosphere cells.

**Figure S36.** The in vitro cytotoxicity of different CPT formulations against BCSC-enriched 4T1 mammosphere cells.

**Figure S37.** The expression of ALDH1 marker in BCSC-enriched 4T1 mammoshpere cells after treatments of different PTX formulations observed by CLSM, scale bar= 100 μm.

**Figure S38.** The expression of ALDH1 marker in BCSC-enriched 4T1 mammoshpere cells after treatments of different DTX formulations observed by CLSM, scale bar=100 μm.

**Figure S39.** The expression of ALDH1 marker in BCSC-enriched 4T1 mammoshpere cells after treatments of different GEM formulations observed by CLSM, scale bar=100 μm.

**Figure S40.** The expression of ALDH1 marker in BCSC-enriched 4T1 mammoshpere cells after treatments of different CPT formulations observed by CLSM, scale bar=100 μm.

**Figure S41.** Tumor images from different treatment groups. (A) NS-treatment group; (B) DOX-treatment group; (C) HA-b-PCDA NP-treatment group; (D) DOX/HA-b-PCDA NP-treatment group.

**Figure S42.** Immunofluorescent staining of CD8^+^ cells in the tumor tissue at the end of the treatment. Blue, cell nucleus; Green, CD8, scale bar=200 μm.

**Figure S43.** Images of H&E staining of major tissues at the end of different treatment groups. (A) NS-treatment group; (B) DOX-treatment group; (C) HA-b-PCDA NP-treatment group; (D) DOX/HA-b-PCDA NP-treatment group. scale bar=20 μm.

**4. Supplementary Tables**

| **Polymer** | **Mn_-theory_ (Da)^a^** | **Mn_-GPC_ (Da)^b^** | **PDI^c^** |
| --- | --- | --- | --- |
| HA | / | 8194 | 1.94 |
| PCDA | / | 3627 | 1.48 |
| HA-b-PCDA | 11821 | 10492 | 2.69 |

## Table S1 Molecular weight data of intermediate and final products of HA-b-PCDA

a: Mn-theory, Theoretical molecular weight calculation based on the Mn_-GPC_ of HA and PCDA.

b: Mn-_GPC_, Number-average molecular weight determined by GPC

c: PDI, Polydispersity index determined by GPC analysis.

## Table S2. The binding energy and interaction type of HA-b-PCDA with related drugs

| **Drug** | **Binding energy**  **(kcal mol^-1^)** | **Hydrogen Bonds** | **π-stacking** | **Cation-π** | **Amide stacking** | **Hydrophobic Interactions** |
| --- | --- | --- | --- | --- | --- | --- |
| Doxorubicin | -7.1 | Yes | Yes | No | No | Yes |
| Gemcitabine | -6.0 | Yes | Yes | No | Yes | Yes |
| Paclitaxel | -6.7 | Yes | Yes | Yes | Yes | Yes |
| Docetaxel | -6.4 | Yes | Yes | No | Yes | Yes |
| Camptothecin | -6.4 | Yes | Yes | No | No | Yes |
| Sorafenib | -6.9 | Yes | Yes | Yes | Yes | Yes |
| Imatinib | -7.6 | Yes | Yes | Yes | Yes | Yes |
| Irinotecan | -7.5 | Yes | Yes | Yes | Yes | Yes |
| Etoposide | -7.4 | Yes | Yes | No | No | Yes |
| Lapatinib | -7.3 | Yes | Yes | Yes | No | Yes |
| Nilotinib | -8.0 | Yes | Yes | Yes | Yes | Yes |
| Carfilzomib | -6.2 | Yes | Yes | Yes | Yes | Yes |
| Regorafenib | -7.4 | Yes | Yes | Yes | Yes | Yes |
| Enzalutamide | -7.0 | Yes | Yes | Yes | Yes | Yes |
| Formestane | -6.3 | Yes | Yes | No | No | Yes |
| Elacridar | -7.6 | Yes | Yes | No | Yes | Yes |
| Edotecarin | -5.8 | Yes | Yes | Yes | Yes | Yes |
| Exemestane | -6.7 | Yes | Yes | No | No | Yes |
| Bexarotene | -7.6 | Yes | Yes | No | No | Yes |
| Trametinib | -7.8 | Yes | Yes | Yes | Yes | Yes |
| Vatalanib | -6.9 | Yes | Yes | Yes | No | Yes |
| Eribulin | -6.9 | Yes | Yes | No | No | Yes |
| Teniposide | -6.9 | Yes | Yes | No | No | Yes |
| Testolactone | -6.8 | Yes | No | No | No | Yes |
| Vindesine | -7.2 | Yes | Yes | Yes | Yes | Yes |
| Ixabepilone | -6.6 | Yes | Yes | No | Yes | Yes |
| Trabectedin | -6.8 | Yes | Yes | No | No | Yes |
| Homoharringtonine | -6.2 | Yes | Yes | No | No | Yes |
| Vinblastine | -6.3 | Yes | No | Yes | No | Yes |
| Vinblastine | -6.3 | Yes | Yes | Yes | No | Yes |
| Capecitabine | -5.8 | Yes | Yes | No | Yes | Yes |
| Gefitinib | -5.5 | Yes | Yes | No | No | Yes |
| Tamoxifen | -5.6 | Yes | Yes | No | No | Yes |
| Erlotinib | -5.5 | Yes | Yes | No | No | Yes |

## Table S3 Characterization of the prepared nanoparticles

| **Drug formulations** | **Size (nm)** | **PDI** | **Zeta-potential**  **(mv)** | **Drug loading content (%)** | **Encapsulation Efficiency**  **(%)** |
| --- | --- | --- | --- | --- | --- |
| HA-b-PCDA NPs | 173.27 | 0.27 | -44.00 | / | / |
| DOX/HA-b-PCDA NPs | 176.50 | 0.21 | -35.57 | 4.98 | 73.99 |
| PTX/HA-b-PCDA NPs | 122.77 | 0.30 | -40.97 | 5.45 | 83.10 |
| DTX/HA-b-PCDA NPs | 126.20 | 0.29 | -39.07 | 4.91 | 75.12 |
| GEM/HA-b-PCDA NPs | 179.80 | 0.26 | -32.60 | 6.28 | 88.57 |
| CPT/HA-b-PCDA NPs | 122.70 | 0.29 | -38.70 | 6.31 | 89.47 |

## Table S4 List of RT-qPCR primers

| **Primer name** | **Sequence (5'->3')** |
| --- | --- |
| Lats1-Mus-Forward | TTCTTCATGGAGCAGCACGTA |
| Lats1-Mus-Reverse | TGAGATAATCCAACCCGCATCA |
| Lats2-Mus-Forward | GCCTTTTGCCAACGAGTCAG |
| Lats2-Mus-Reverse | CCAGGAATATGCATCTGGTCAC |
| Yap1-Mus-Forward | TCAATGCCGTCATGAACCCC |
| Yap1-Mus-Reverse | GCATCAGTACTGGCCTGTCG |
| Myc-Mus-Forward | CACCAGCAGCGACTCTGAAGAA |
| Myc-Mus-Reverse | TAGTTGTGCTGGTGAGTGGAG |
| Ctgf-Mus-Forward | TGGAGGAAAACATTAAGAAGGGCA |
| Ctgf-Mus-Reverse | TCGGGGCATTTGAACTCCAC |
| Id1-Mus-Forward | AGGATCATGAAGGTCGCCAG |
| Id1-Mus-Reverse | AGCCGTTCATGTCGTAGAGC |
| Id2-Mus-Forward | ACTCGCATCCCACTATCGTC |
| Id2-Mus-Reverse | AAGGGAATTCAGATGCCTGCAA |
| Ctnnb1-Mus-Forward | TGCAGCGACTAAGCAGGAAG |
| Ctnnb1-Mus-Reverse | AAGGACGGTGCGTACAAGAG |
| Jak2-Mus-Forward | GGAGATTATGGTCAACTGCACAA |
| Jak2-Mus-Reverse | GCTGCTTCGAAGAAAGACTCTGA |
| Stat3-Mus-Forward | CCTCTACCCCGACATTCCCA |
| Stat3-Mus-Reverse | GTATTGCTGCAGGTCGTTGGTG |
| Bcl2-Mus-Forward | GGATAACGGAGGCTGGGATGC |
| Bcl2-Mus-Reverse | CACTTGTGGCCCAGGTATGC |
| Gfap-Mus-Forward | GGAGTCGGCCAGTTACCAG |
| Gfap-Mus-Reverse | ACAGGAATGGTGATGCGGTTT |
| Aldh1a1-Mus-Forward | GTCGGATTTAGGAGGCTGCATA |
| Aldh1a1-Mus-Reverse | GGCCACACACTCCAATAGGT |
| Oct4-Mus-Forward | GGGCGTTCTCTTTGGAAAGGTG |
| Oct4-Mus-Reverse | TGCTAGTTCGCTTTCTCTTCCG |
| Nanog-Mus-Forward | TGAGCTATAAGCAGGTTAAGAC |
| Nanog-Mus-Reverse | CTGGGATACTCCACTGGTGC |
| Epcam-Mus-Forward | GATCCAGAACAACGATGGGC |
| Epcam-Mus-Reverse | TCAATGATGATCCAGTAGGTCC |
| Sox2-Mus-Forward | TTTGTCCGAGACCGAGAAGC |
| Sox2-Mus-Reverse | CTCCGGGAAGCGTGTACTTA |
| Csf1-Mus-Forward | GTAGACCAGGAACAGCTGGATG |
| Csf1-Mus-Reverse | AGAGTTCCTGGAGCCTCTCG |

## Table S5 Antibodies

| **Primary antibody** | **Host** | **Dilution** | **Source** | **Catalog Number** |
| --- | --- | --- | --- | --- |
| anti-LATS1/2 | Rabbit | 1:1000 | Cohesion | CPA7180 |
| anti-phospho- LATS1/2 | Rabbit | 1:1000 | SAB | 11736 |
| anti-YAP1 | Rabbit | 1:1000 | Abclonal | A19134 |
| anti-phospho-YAP1 | Rabbit | 1:1000 | Bioss | bsm-52214R |
| anti-c-MYC | Rabbit | 1:1000 | Abclonal | A19032 |
| anti-CTGF | Rabbit | 1:1000 | Boster | PB0570 |
| anti-Id1 | Rabbit | 1:1000 | SAB | 45023-1 |
| anti-JAK2 | Rabbit | 1:1000 | Boster | BM4165 |
| anti-phospho-JAK2 | Rabbit | 1:1000 | Boster | BM4839 |
| anti-STAT3 | Rabbit | 1:1000 | Boster | BM4052 |
| anti-phospho-STAT3 | Rabbit | 1:1000 | Boster | BM4835 |
| anti-ALDH1A1 | Rabbit | 1:500 | Bioss | bsm-60321R |
| anti-OCT4 | Rabbit | 1:500 | Bioss | bsm-52002R |
| anti-NANOG | Rabbit | 1:500 | Bioss | bsm-52124R |
| anti-EPCAM | Rabbit | 1:1000 | Bioss | bsm-52417R |
| anti-β-ACTIN | Rabbit | 1:3000 | Beyotime | AF5003 |
| anti-GAPDH | Rabbit | 1:3000 | Boster | BM3874 |
| anti-Lamin B1 | Mouse | 1:3000 | Proteintech | 66095-1-Ig |
| anti-CD8a | Rabbit | 1:100 | Abclonal | A23081 |
| **Secondary antibody** |  |  |  |  |
| HRP-conjugated goat anti-Rabbit IgG | Goat | 1:2000 | Beyotime | A0208 |
| HRP-conjugated goat anti-Mouse IgG | Goat | 1:5000 | Bioss | bsm-40296G |
| Alexa Fluor® 488-conjugated goat anti-Rabbit IgG | Goat | 1:200 | CST | 4412S |

**References**

1. Lv L, Guo Y, Shen Y, Liu J, Zhang W, Zhou D, and Guo S. Intracellularly Degradable, Self-Assembled Amphiphilic Block Copolycurcumin Nanoparticles for Efficient In Vivo Cancer Chemotherapy. Advanced Healthcare Materials. 2015; 4(10): 1496-501.https://doi.org/10.1002/adhm.201500075.

2. Huang J, Zhang H, Yu Y, Chen Y, Wang D, Zhang G, Zhou G, Liu J, Sun Z, Sun D, Lu Y, and Zhong Y. Biodegradable self-assembled nanoparticles of poly (D,L-lactide-co-glycolide)/hyaluronic acid block copolymers for target delivery of docetaxel to breast cancer. Biomaterials. 2014; 35(1): 550-66. https://doi: 10.1016/j.biomaterials.
